# Supplementary material for: Patterns of sexual violence against adults and children during the COVID-19 pandemic in Kenya: a prospective cross-sectional study
Source: BMJ Open. 2021 Sep 6;11(9):e048636. doi: 10.1136/bmjopen-2021-048636 (PMC8423516; doi:10.1136/bmjopen-2021-048636)
Supplement: Supplementary data [file bmjopen-2021-048636supp001.pdf]

SURVIVORS DATA COLLECTION INTAKE SUMMARY FORM

a. Date of incident: Time of incident:
b. Age of survivor: Sex:
c. Location(s) of incident:
   - Survivor's house: **Yes/No**
   - Other's house: **Yes/No**
   - Place of Work: **Yes/No/I don't know**
   - Perpetrator's house: **Yes/No/I don't know**
   - On my way to work/ On the road: **Yes/No/I don't know**
   - Other location (please describe): **Yes/No/I don't know**
d. Name of the city/town/village where the incident took place:
e. Brief description of the incident:

f. Perpetrator's Information: -
   - Number of perpetrator(s): - Do you know the perpetrator(s): -
   - If Yes, How
   - What is the gender: - Age of the perpetrator: -
g. Post Incident Information: -
   - What action did the survivor take after the incident?
     a. Seek medical attention: **Yes/No**
     b. Seek counselling: **Yes/No**
     c. Report to the police: **Yes/No**
     d. Seek legal redress: **Yes/No**
     e. Protection/Safe space/Shelter: **Yes/No**
h. If the survivor sought medical attention for the incident:
   a. Did they become HIV positive due to the incident? **Yes/No/I don't know**
   b. Did you get pregnant due to the incident? **Yes/No/I don't know**
   c. Did you contract any STI's due to the incident? **Yes/No/I don't know**
   d. Were you physically injured due to the incident? **Yes/No/I don't know**
i. Was the survivor referred to the WKF? Yes/No If **YES:** Client's Ref. No:
