# Supplementary material for: Patterns of sexual violence against adults and children during the COVID-19 pandemic in Kenya: a prospective cross-sectional study
Source: BMJ Open. 2021 Sep 6;11(9):e048636. doi: 10.1136/bmjopen-2021-048636 (PMC8423516; doi:10.1136/bmjopen-2021-048636)
Supplement: Author's manuscript [file bmjopen-2021-048636.draft_revisions.pdf]

BMJ Open is committed to open peer review. As part of this commitment we make the peer review history of every article we publish publicly available.

When an article is published we post the peer reviewers' comments and the authors' responses online. We also post the versions of the paper that were used during peer review. These are the versions that the peer review comments apply to.

The versions of the paper that follow are the versions that were submitted during the peer review process. They are not the versions of record or the final published versions. They should not be cited or distributed as the published version of this manuscript.

BMJ Open is an open access journal and the full, final, typeset and author-corrected version of record of the manuscript is available on our site with no access controls, subscription charges or pay-per-view fees (http://bmjopen.bmj.com).

If you have any questions on BMJ Open's open peer review process please email info.bmjopen@bmj.com

BMJ Open

Patterns of Violence Against Adults and Children During the COVID-19 Pandemic in Kenya

Journal:	*BMJ Open*
Manuscript ID	bmjopen-2021-048636
Article Type:	Original research
Date Submitted by the Author:	05-Jan-2021

Complete List of Authors:	Rockowitz, Sarah; University of Birmingham, School of Psychology<br>Stevens, Laura M.; University of Birmingham, School of Psychology<br>Rockey, James; University of Birmingham, Birmingham Business School<br>Smith, Lisa; University of Leicester, Criminology<br>Richie, Jessica; University of Leicester<br>Colloff, Melissa; University of Birmingham, School of Psychology<br>Kanja, Wangu; Wangu Kanja Foundation<br>Cotton, Jessica; University of Birmingham, School of Psychology<br>Flowe, Heather; University of Birmingham, School of Psychology

Keywords:	COVID-19, PUBLIC HEALTH, Child protection < PAEDIATRICS

SCHOLARONE™
Manuscripts

I, the Submitting Author has the right to grant and does grant on behalf of all authors of the Work (as defined in the below author licence), an exclusive licence and/or a non-exclusive licence for contributions from authors who are: i) UK Crown employees; ii) where BMJ has agreed a CC-BY licence shall apply, and/or iii) in accordance with the terms applicable for US Federal Government officers or employees acting as part of their official duties; on a worldwide, perpetual, irrevocable, royalty-free basis to BMJ Publishing Group Ltd ("BMJ") its licensees and where the relevant Journal is co-owned by BMJ to the co-owners of the Journal, to publish the Work in this journal and any other BMJ products and to exploit all rights, as set out in our licence.

The Submitting Author accepts and understands that any supply made under these terms is made by BMJ to the Submitting Author unless you are acting as an employee on behalf of your employer or a postgraduate student of an affiliated institution which is paying any applicable article publishing charge ("APC") for Open Access articles. Where the Submitting Author wishes to make the Work available on an Open Access basis (and intends to pay the relevant APC), the terms of reuse of such Open Access shall be governed by a Creative Commons licence – details of these licences and which Creative Commons licence will apply to this Work are set out in our licence referred to above.

Other than as permitted in any relevant BMJ Author's Self Archiving Policies, I confirm this Work has not been accepted for publication elsewhere, is not being considered for publication elsewhere and does not duplicate material already published. I confirm all authors consent to publication of this Work and authorise the granting of this licence.

For peer review only

Patterns of Violence Against Adults and Children During the COVID-19 Pandemic in Kenya

Sarah Rockowitz[1], Laura M. Stevens[1], James Rockey[1], Lisa Smith[2], Jessica Richie[2], Melissa Colloff[1], Wangu Kanja[3], Jessica Cotton[1], and Heather Flowe[1]

Corresponding author:
Heather Flowe
School of Psychology, University of Birmingham
52 Pritchatts Road, Birmingham, UK B15 2SA
h.flowe@bham.ac.uk

University of Birmingham, School of Psychology, Birmingham, UK [1]
University of Leicester, School of Criminology, Leicester, UK[2]
The Wangu Kanja Foundation, Nairobi, Kenya[3]

Keywords: Gender-based violence, sexual abuse of child, rape, Kenya, exposure to violence

Word count: 4,098

For peer review only

Author Note

We thank the Survivors of Sexual Violence Network in Kenya and the Social Justice Network Group for collecting the data for this study, and for the survivors who shared their stories with us.

Our data are available (https://osf.io/bqrn2/?view_only=4536951b3a9640078649b2e4cdfc956f).

Sections of these data were included in a preliminary report and policy brief (https://zenodo.org/record/3964124#.X-OVdGacYWo; and https://zenodo.org/record/3964162#.X-OVk2acYWo)

For peer review only

ABSTRACT

Objectives This study examined patterns of sexual violence against adults and children in Kenya during the COVID-19 pandemic to inform sexual violence prevention and protection efforts.

Design A prospective cross-sectional research design was used.

Setting Kenya

Participants 317 adults, 224 children

Main Measures Perpetrator and survivor demographic data, characteristics of the assault.

Results Children were more likely than adults to be attacked during the daytime, by a single perpetrator rather than multiple perpetrators, and in a private as opposed to a public location. Children were violated most often by neighbors and family members, whereas adults were equally likely to be attacked by strangers and persons known to them. On average, the children in the sample were four years younger compared to the average age reported in national samples pre-pandemic (age 12 versus 16). Survivors were more likely to be female than male.

Conclusions Patterns of sexual violence against adult and child survivors during the COVID-19 pandemic are different, suggesting age-related measures are needed in national emergency plans to adequately address sexual violence during the pandemic.

For peer review only

Strengths and limitations of this study

This study was conducted in partnership with frontline, survivor-led organisations, using a prospective study design, which enabled the systematic and rapid study of sexual violence in Kenya during the pandemic, even though there were considerable physical distancing measures in place.

The data provide detailed information about survivors and perpetrators, including where and when incidents occurred, which enabled us to compare patterns of sexual violence in adults and children.

The sample was comprised of individuals who were seeking help in accessing vital services; therefore, inferences about patterns nationally in Kenya cannot be made because the data may not be a representative.

Information about whether patterns of sexual violence are changing during the pandemic remains unknown because sexual violence is underreported, and there is a need for real time data collection systems that gather and analyse detailed, longitudinal information about sexual violence incidents in low- and middle-income countries like Kenya.

For peer review only

Patterns of Violence Against Adults and Children During the COVID-19 Pandemic in Kenya

Sexual and gender-based violence (SGBV) affects women in all societies. Nearly a third of ever-partnered women worldwide have experienced physical and/or sexual intimate partner violence at least once in their lives, and 7.2% of all women have experienced non-partner sexual violence.[1] This study focuses on Kenya, a country that has a long history of SGBV, which is exacerbated during times of national crisis, such as during election periods.[2] Previous conflicts and disasters have led to increased gender inequality, gender-based violence and other human rights violations, owing to disruptions in medical, protection, and legal services.[3] The arrival of COVID-19 in Kenya in early March 2020 marked the start of another national crisis, with more than 56,000 Kenyans becoming sick as of November 2020.[4-6] In late March, President Kenyatta issued a nation-wide curfew, with all non-essential travel banned between 7pm to 5am. Schools and non-essential businesses had to close, and travel in and out of the country was heavily restricted.[5, 6] These measures have been extended and modified multiple times, and as of November 2020, the number of people who can gather in groups is still limited. Schools are still closed, while universities have been opened, and air travel restrictions have been lifted.[6] While the measures have undoubtedly curbed the spread of the disease, they seem to be compromising the safety and well-being of citizens. In particular, there have been widespread reports of increases in domestic and sexual violence during the COVID-19 crisis.[6, 7] Indeed, there is worldwide concern that the pandemic will exacerbate gender inequality, and negatively impact the care and treatment women and girls receive in the aftermath of violence, owing to decreased access and funding following COVID-19 economic shocks.[8]

Around the world, humanitarian crises, such as natural disasters, conflict, and disease outbreaks, are associated with changing patterns of sexual violence.[9-12] After the 2010 earthquake in Haiti, for example, the odds of an adolescent girl in Haiti being sexually abused increased by 41%.[11] There have also been documented increases of violence against women after floods, hurricanes, and earthquakes in the United States, Canada, and Australia, as well as following the 2004 tsunami in Sri Lanka, and droughts in Ethiopia, Zimbabwe, and Kenya.[13, 14] Increased sexual violence occurs during conflicts, notably in Rwanda, Kosovo, and the Democratic Republic of the Congo (DRC). Sexual violence in these contexts has been used as a means of ethnic cleansing against the Tutsis, Kosovars, and Congolese women by the Hutus, Serbians, and Congolese soldiers, respectively.[15-18] These crimes are especially prevalent against women and children, and attacks by multiple perpetrators are common. In the DRC, for example, nearly 76% of women surveyed had experiences of rape that were consistent with the attack being used as a weapon of war, and 69% of women reported experiencing gang rape, with these incidents typically being perpetuated by three perpetrators on average.[17] These findings are consistent with research conducted in the Central African Republic, Libya, and Mali, which found that multiple-perpetrator rape was commonly reported by survivors.[19]

SGBV increases during disease outbreaks, with studies reporting increases in Sierra Leone, Liberia, and Guinea during the Ebola outbreaks in West Africa in 2014-2016, and especially high increases in teenage pregnancies were reported in Sierra Leone.[12, 20] Similarly, Zika and cholera outbreaks have been linked with increased incidence of domestic violence, and reductions in funding for and access to public health services.[21] Physical distancing measures implemented during pandemics are also thought to be responsible for changing patterns and increases in violence. For instance, lockdowns and curfews mean that people have to remain

For peer review only

indoors with abusers and are unable to access outside assistance because police and vital services are unavailable, and abusers can act with impunity.[22,23]

More research on SGBV during times of compounding crises is needed, however. Gender-based violence is highly stigmatized, which leads to underreporting, especially in developing countries that have high levels of gender inequality. Further, it is difficult to assess whether patterns and rates of SGBV are changing during times of crisis, owing to the unavailability of nationally representative data and a lack of up-to-date and recurring data collection, which would allow for examining SGBV trends in relation to humanitarian crises, and inform effective prevention, protection and responses.

During the current COVID-19 outbreak, seven months of lockdown measures, economic challenges, health concerns, and changing global relations have increased concerns that women have a heightened risk of SGBV. This violence during lockdown is being considered a shadow pandemic with the UN Population Fund estimating an additional 31 million cases of SGBV worldwide following 6-months isolation.[23, 24] This increased risk of violence and abuse for women is coupled with other gendered impacts of the pandemic, including the gendered nature of healthcare (e.g., 70% of the healthcare and social services workforce globally are women) putting women at increased chance of exposure, increased caregiving responsibilities, and a loss of informal sector work and therefore financial freedom.[25] Governments in some countries, for example, have had to create or supply alternate housing for people fleeing abusive situations, as was the case with in Italy and France, with hotels being used as safe houses.[26] Social isolation policies have distinct impacts on children as well. Adolescent girls' absence from school, coupled with the lack of alternative safe venues, has been associated with increased vulnerability to sexual violence from family members and others, including neighbours and

For peer review only

community members.[27] As seen during the Ebola crisis, the closure of schools was associated with increased sexual violence against girls, adolescent pregnancy, and adolescent marriage.[28]

This study prospectively investigated patterns of sexual violence perpetrated against adults and children in Kenya during the COVID-19 pandemic. We analyse data from interviews with adult survivors and the guardians of child survivors conducted by human rights defenders during the pandemic. We focus on sexual violence because it has received less attention during the pandemic compared to other forms of gender-based violence. Further, research to date has not compared patterns of violence for adults and children. Doing so is critical because social isolation measures may differentially affect people in relation to age, and different measures may need to be put in place depending on the age group to prevent and respond to SGBV during COVID-19.

Based on the literature reviewed above, we predicted that there would be a greater number of women and girls violated compared to men and boys. Additionally, we anticipated there would be age-related differences in the types of locations in which sexual violence is occurring. Owing to school closures, and a lack of alternative safe spaces, we predicted that children would be at a greater risk than adults during the day, in private compared to public locations. We also compared the incidence of multiple versus single perpetrator attacks to better understand the nature of the violence occurring in relation to age. To our knowledge, no previous research has compared adults and children regarding the prevalence of violations committed by multiple perpetrators. Hence, no age group predictions were made concerning multiple perpetrators.

METHOD

Design

For peer review only

A quantitative between groups prospective research design was used. The criterion variable was age group (child or adult survivor). The predictor variables included the offense characteristics displayed in Table 1, which also summarises how the variables were operationalized.

For peer review only

Table 1

Descriptions of how predictor variables were coded and operationalised

Variable	Definition
Female Survivor	Whether the survivor was female (coded as 1) as opposed to male (coded as 0)
Male Perpetrator	Whether the perpetrator was male (coded as 1) as opposed to female (coded as 0)
Daytime Attack	Whether the attack occurred in the daytime (6:00am-5:59pm; coded as 1) as opposed to at night (6:00pm-5:59am; coded as 0)
Private or Public Location	Whether the attack occurred in a private home (coded as 1) as opposed to a public location where the violation could have been witnessed or interrupted by a member of the public (coded as 0).
Private Location Type	Private locations were further subdivided into victim residence (coded as 1 for victim residence, 0 for any other location public or private); perpetrator residence (coded as 1 for perpetrator residence, 0 for any other location public or private); or other residence (coded as 1 for other residence, 0 for any other location public or private).
Multiple Perpetrator	Whether the attack was perpetrated by more than one perpetrator (coded as 1) as opposed to a singular perpetrator (coded as 0).
Known or Stranger Perpetrator	Whether the attack was perpetrated by someone known to the survivor (coded as 1) or a stranger (coded as 0)
Perpetrator Relationship Type	Perpetrator relationship type was subdivided into neighbour (coded as 1 for neighbour, 0 for any other relationship type); stranger (coded as 1 for stranger, 0 for any other relationship type); family member (coded as 1 for family member, 0 for any other relationship type); acquaintance/friend (coded as 1 for acquaintance/friend, 0 for any other relationship type); spouse/husband/boyfriend (coded as 1 for spouse/husband/boyfriend, 0 for any other relationship type); authority figure (coded as 1 for authority figure, 0 for any other relationship type); or other (coded as 1 for other, 0 for any other relationship type).

For peer review only

Participants

Participants (*N*=787) were survivors of sexual violence. All were residents of Kenya, living in 23 counties, and had contacted human rights defenders for assistance in obtaining vital services in the aftermath of sexual violence during the COVID-19 pandemic. The human rights defenders interviewed the survivors (or their legal guardians if they were under 18) about the offense upon intake. The interview protocol was informed by WHO ethical principles for research on SGBV and safety protocols developed by the human rights defenders for conducting their work with survivors.[29] The survivors were aged between 7 months and 72 years (*M* = 21.3; *SD* = 9.4).

Survivors were categorized into two age groups. Following definitions provided by WHO, the child group included survivors aged 17 years and younger, whereas the adult group included survivors aged 18 years and older.[1]

After excluding cases with missing data on the predictor variables, the final sample consisted of 224 survivors in the child group and 317 in the adult group. The participants in the final sample for the child group ranged in age from 8 months to 17 years (*M* = 12.6*, SD* = 3.9), 83% were girls, and 93% were perpetrated against by men, and for the adult group, ranged in age from 18 to 72 (*M* = 27.1*, SD* = 8.1), 92% were women, and 96% were perpetrated against by men.

Materials

The data were obtained from records held by the human rights defenders who were assisting survivors in accessing vital services during the pandemic. They interviewed survivors about the incident and recorded information about the case on their standard intake form. They recorded the date, time, and location of the incident, and gave a free text description summarising the incident. The form also had specific items to document the number of perpetrators, the relationship between the survivor and perpetrator(s), the location of the attack, and the age and gender of the survivor

For peer review only

and perpetrator. Additionally, whilst not analysed in the current paper, any services (e.g., police, medical, safe house) the survivor had accessed were also recorded.

Procedure

Each intake form was read by two members of the research team to create the dataset. They coded the data using the criteria outlined in Table 1. If there was missing data on the form, the team read the incident summary and attempted to complete the missing information.

Ethics

The confidentiality of the data was maintained by the research team, and participants were not placed at risk of harm as a result of this study. The Kenyan Data Protection Act (2019) was adhered to in the conduct of this research study.[30] The data belong to the Wangu Kanja Foundation and the Sexual Violence Survivors' Network in Kenya, and permission to use the data was obtained from these organisations to conduct the analyses. The research was also approved by the STEM Ethics Committee at the University of Birmingham.

Patient and Public Involvement

We relied heavily on input from civil society grassroots organisations who work on the frontlines to assist suvivors in accessing vital services in the aftermath of sexual violence, including the Wangu Kanja Foundation and the Sexual Violence Survivors' Network in Kenya. These organisations co-developed of the research questions, the study design, including the data collection instruments, as well as participant recruitment, data collection, and manuscript preparation. Their experience and knowledge with sexual violence in Kenya informed every aspect of the project. Their reputation within the Kenyan communities enabled survivors to disclose the incidents that occurred. The Wangu Kanja Foundation and Human Rights Defenders

For peer review only

will be integral in disseminating the research findings to their networks and relevant stakeholders.

Statistical analysis

As our main analysis, we used logistic regression with age group as the dependent variable to determine which offense characteristics significantly differentiated the child and adult groups. The child age group was coded as 1 in the analysis, whereas the adult age group was coded as 0. While our data contain detailed information about each attack we restrict our analysis to a limited number of binary variables in Table 1 as predictors. This is because there is a risk of a common-support problem if we use a finer grained analysis. For example, whilst we have detailed data on where survivors were attacked, or relationship with the perpetrator, we cannot exploit this as few in the child group were attacked going to work, or by their spouse or partner. To avoid this difficulty, we used the coarser coding of relationship, known versus stranger perpetrator, and whether the attack took place in a public place or in private. This ensured that there were sufficient numbers of both children and adults in all categories. We then supplement this analysis by tabulating the finer coding in Tables 2 and 3 respectively.

Table 2

Distribution of Perpetrator Relationship to Survivor within Age Group

	Child<br>*n*=224	Adult<br>*n*=317
Neighbour	29%	6%
Stranger	25%	41%
Family member	20%	5%
Other	12%	16%
Acquaintance/friend	11%	12%
Spouse/husband/boyfriend	3%	15%
Authority figure	2%	6%

For peer review only

Table 3
Distribution of Attack Location within Age Group

	Child *n*=224	Adult *n*=317
Perpetrator's house	41%	20%
Public	28%	48%
Survivor's house	23%	23%
Other house	7%	6%
Survivor/perpetrator's house	1%	3%

We conducted preliminary analyses to identify which variables to enter into the model using Pearson's chi square tests for association. This allowed for testing whether the association between age group and each of the dichotomous variables was statistically significant. Only the variables that were significantly associated with age group were entered into the logistic regression model. To control for Type 1 errors, Bonferroni corrections were applied to the .05 alpha level (adjusted alpha = .008, with 6 variables). The strength of the relationship between the individual offense characteristics was assessed using Cramer's V, which measures the magnitude of the relationship between two categorical variables.[31] Values that fall between .40 and .60 are interpreted as large, whereas values that fall between .20 and .40 are interpreted as moderate in magnitude. Values smaller than .20 are regarded as associations that are small in magnitude. All analyses were conducted using SPSS version 26.

Our data are freely available: https://osf.io/b9dzp/.

RESULTS

The results of the chi square analysis are presented in Table 4. Results indicate that children compared to adults were less likely to be female and less likely to be attacked by multiple perpetrators. Children compared to adults were more likely to be attacked in a private location, by

For peer review only

a known perpetrator and were more likely to be attacked in the daytime. The association between age and private location, and age and multiple perpetrators was moderately large, whereas the strength of the other associations, while statistically significant, was small in magnitude.

Table 4
Chi Square Outputs

	Child	Adult	Pearson's Chi Square	*p*	Cramer's V
Variable	*n*=224	*n*=317			
Female victim	83%	92%	11.41	.001	.145
Male Perpetrator	92%	94%	1.17	.279	.047
Day	59%	44%	13.18	<.001	.156
Private versus Public Location	66%	45%	21.55	<.001	.200
Multiple	13%	31%	24.20	<.001	.212
Known versus Stranger Perpetrator	76%	58%	17.86	<.001	.182

In the logistic regression model, the variables that were statistically significant from the chi square results were entered as predictors (i.e., female survivor, daytime attack, private versus public location, multiple perpetrators, and known versus stranger perpetrator), and the dependent variable was age group. The overall model was statistically significant, x^2 (5, N = 541) 53.3, p = < .001. According to Nagelkerke's R^2, 13% of the variability in age group was accounted for by the predictors in the model. Predictive accuracy improved from 59% to 64% using the model. The variables in our model were able to correctly classify 78% of cases perpetrated against adults, whereas 44% of cases in the child group were correctly classified using the variables in our model.

For peer review only

As shown in Table 5, all of the variables, except whether the crime was committed by a known perpetrator, were statistically significant. Child compared to adult survivors were 1.61 times more likely to be attacked during the day, and 1.72 times more likely to be attacked in private as opposed to in public. Child compared to adult survivors were also significantly less likely (*OR*=.458) to be female, and less likely (*OR*=.528) to be attacked by multiple perpetrators.

Table 5
Table of coefficient

	df	Estimate	SE	Wald X^2	*p*
Female victim	1	-0.782	0.29	7.3	.007
Day	1	0.474	0.19	6.5	.011
Private versus Public Location	1	0.543	0.21	6.96	.008
Multiple	1	-0.638	0.27	5.77	.016
Known versus Stranger Perpetrator	1	0.295	0.34	1.6	.21

Tables 2 and 3 present a more detailed descriptive analysis of the child and adult cases on the relationship between the perpetrator and the victim, and the locations in which the attacks took place. As can be seen, age group was significantly associated with the relationship between the perpetrator and the survivor, X^2 (7, N = 541) = 107.84, p < .001. Children were most often victimized by neighbors, followed by strangers and family members, whereas adults were most often victimized by strangers followed by other types of perpetrators (customer, community member, friend of a friend) and spouses. Age group was also significantly associated with attack location, X^2 (4, N = 541) = 35.59, p < .001. Children were more most often attacked at the

For peer review only

perpetrator's house (41% of the cases), whereas adults were most often attacked in public locations (48% of cases).

DISCUSSION

Summary of Key Findings

We compared patterns of sexual violence committed against adults and children in Kenya during the COVID-19 pandemic. The data arose from interviews conducted by human rights defenders with survivors, and describe the experiences of 541 survivors. We found that the children in our sample were on average four years younger compared to national surveys of children in Kenya. [32, 33] Further, compared to adults, children were more likely to be attacked during the day, in private as opposed to public locations, by lone perpetrators, and by neighbors. In what follows, we discuss our findings in relation to existing research and draw implications for policy.

Comparisons to Current Literature

There were significant numbers of children in our sample, which is unsurprising, as approximately half of GBV survivors are children during humanitarian crises. However, the children in our sample were 12 years old on average, which is 4 years younger than the nationally representative samples taken pre-pandemic.[32, 33]

We also found that children were 1.61 times more likely than adults to be attacked during the day. This could be attributed to the way that children and adults were spending their time during the pandemic. Because schools were closed, and there was no provision of any alternative safe spaces, children may have been often left alone or under the care or supervision of neighbors or community members, which may have made them more vulnerable to attack in some instances. Children were more likely to be attacked in private as compared to public locations. Adults in

For peer review only

our sample were about equally likely to be attacked during the day as at night. Further, in keeping with previous research, significantly more adults were violated by multiple perpetrators compared to children.[36]

The proportion of boys in the children's group was larger than the proportion of men in the adult's group. This may reflect differential rates of victimization for men compared to boys. Another possible reason is that sexual violence against men compared to boys is disclosed less often. The legal definition of rape in Kenya, like many countries, requires 'vaginal penetration', which reinforces sociocultural notions that men cannot be sexually victimised.[34] Further, the tendency for people to believe that the victimisation of men is harmless, coupled with self-blame and fear on the part of victims that their community and family will react negatively towards them, discourage men from seeking help, and reporting sexual offenses to the police.[35]

The children and adults in our sample were more likely to be violated by someone they knew than a stranger. For adults, perpetrators were most likely to be strangers, followed by neighbors and community members, and spouses. The most common perpetrators for children were neighbors, followed by strangers, and family members. Adults were violated by strangers more frequently because they were often attacked when the opportunity struck, such as while walking to or from work, whereas children were violated by neighbors when they were left under their supervision due to school closures and their parents' job requirements.[25] Children were attacked by neighbors and in the perpetrators' houses at higher rates than adults. Although both groups were more likely to be violated by someone they knew as opposed to a stranger, and in both groups more than half of the perpetrators were known to the survivor, there is a high proportion of stranger compared to known assailants in both age groups. There were a number of instances

For peer review only

in our data set in which neighbors invited children to use a computer or access the Internet, and then assaulted them once they were inside the neighbor's residence.

Strengths and Limitations

This research was conducted in partnership with frontline, survivor-led organisations, using a prospective study design, which are key strengths. This enabled us to systematically and rapidly study sexual violence in Kenya during the pandemic, even though there were considerable physical distancing measures in place. Further, our data are unusually rich. The data provide detailed information about survivors and perpetrators, where and when the incidents occurred, which allowed for studying patterns of sexual violence. There are also several limitations of our study to note. First, the sample was comprised of individuals who were seeking help in accessing vital services. Hence, inferences about patterns nationally in Kenya cannot be made because the data may not be a representative. Further, our data do not provide information about sexual violence trends. Like many countries around the world, sexual violence is underreported, and detailed, longitudinal information about sexual violence incidents are lacking. Consequently, researchers struggle to make inferences about whether patterns of violence are changing during COVID-19.[37] For example, the Demographic and Health Survey in Kenya, which is a nationally representative survey of adults occurring every five years, does not gather in-depth information about violations. Similarly, the national Violence Against Children Survey in Kenya, conducted in 2010 and 2019, does not gather in depth information. We also had to rely on second-hand accounts from guardians of sexual violence against children, owing to a lack of trained personnel and adequate resources in Kenya for interviewing children. Some children were too young to be interviewed, with the youngest victim being just seven-months old. Finally, our model better accounted for patterns of violence against adults compared to children. This is because some of

For peer review only

the factors we analysed were more applicable to adults than children (e.g., employment, romantic relationships, being alone in public).

Recommendations and Conclusion

We urge policy makers to make sure that government COVID-19 emergency management and recovery planning adequately covers sexual and gender-based violence. This should include the provision of adequate alternative safe venues when schools are closed. Further, the implementation of emergency routes that enable survivors to access health, security, and protection services need to be a part of government plans. Survivors should also be exempted from lockdown requirements so they can access services. Further, the medico-legal response to SGBV can be strengthened by expediting restraining orders and prosecutions, and by establishing 'one-stop' centres to allow survivors to access essential services, and authorities to collect evidence, all in one location. In conclusion, governments must invest in real-time data collection and analysis systems to capture the nature of current sexual violence incidents and study regional trends. This would allow authorities to identify crime hot spots and violations being perpetrated by the serial offenders, and to monitor the accessibility of vital services to help ensure that survivors have support. These efforts should be survivor-centred, and involve survivors in the implementation and evaluation of the systems.

.

For peer review only

Declarations

Authors' Contributions

HF and WK conceptualized and designed the data collection methods utilized in this project. SR and LMS drafted the protocol which was then reviewed by HF, MC, JR, and LS. All authors contributed to the reviewed draft version of the manuscript and approved the final version.

Competing interests

The authors declare that they have no competing interests.

Funding

This research was funded by ESRC Grant ES/T010207/1 (to H.D.F., M.F.C., J.C.R. and W.K.F.), the Institute for Global Innovation, University of Birmingham (to H.D.F, and W.K.F.), and a research grant from the Ring for Peace Foundation (to L.S.).

Ethics

This research was approved by the University of Birmingham Ethics Committee (ERN_19-0183).

Availability of data and materials

All data generated during this study will be included in the published scoping review and will also be made available upon request.

For peer review only

References

1. Garcia-Moreno C, Pallitto C, Devries K, Stockl H, Watts C, Abrahams N. Global and regional estimates of violence against women: prevalence and health effects of intimate partner violence and nonpartner sexual violence. Geneva: WHO; 2013.
2. Odhiambo A. "They Were Men in Uniform" Sexual Violence Against Women and Girls in Kenya's 2017 Elections USA: Human Rights Watch 2017.
3. Bellizzi S, Nivoli A, Lorettu L, Ronzoni AR. Human rights during the COVID-19 pandemic: the issue of female genital mutilations. Public health (London). 2020;185:53-4.
4. Health Mo. First Case of Coronavirus Disease Confirmed in Kenya health.go.ke: Ministry of Health; 2020 [
5. Ritchie H, Ortiz-Ospina E, Beltekian D, Mathieu E, Hasell J, Macdonald B, et al. Kenya: Coronavirus Pandemic Country Profile ourworldindata.org: ourworldindata.org; 2020 [
6. Flowe H, Rockowitz S, Rockey J, Kanja W. Sexual and Other Forms of Violence During the COVID-19 Pandemic Emergency in Kenya. The University of Birmingham 2020.
7. Bearak M, Ombuor R. Kenya's coronavirus curfew begins with wave of police crackdowns. The Washington post. 2020.
8. UNFPA. As pandemic rages, women and girls face intensified risks 2020 [Available from: https://www.unfpa.org/news/pandemic-rages-women-and-girls-face-intensified-risks.
9. Marsh M, Purdin S, Navani S. Addressing sexual violence in humanitarian emergencies. Glob Public Health. 2006;1(2):133-46.
10. Dara Kay C, Ragnhild N. Sexual violence in armed conflict: Introducing the SVAC dataset, 1989–2009. Journal of peace research. 2014;51(3):418-28.
11. Sloand E, Killion C, Yarandi H, Sharps P, Lewis-O'Connor A, Hassan M, et al. Experiences of violence and abuse among internally displaced adolescent girls following a natural disaster. J Adv Nurs. 2017;73(12):3200-8.
12. Schwartz DA, Anoko JN, Abramowitz SA. Pregnant in the Time of Ebola Women and Their Children in the 2013-2015 West African Epidemic [text]. Cham: Springer International Publishing : Imprint: Springer,; 2019.
13. Fisher S. Violence Against Women and Natural Disasters: Findings From Post-Tsunami Sri Lanka. Violence Against Women. 2010;16(8):902-18.
14. Le Masson V, Lim S, Budimir M, Podboj J. Disasters and violence against women and girls. Can disasters shake social norms and power relations? London, England: Overseas Development Institute; 2016.
15. Watch HR. Kosovo: Rape as a Weapon of "Ethnic Cleansing. New York, NY; 2000.
16. Lives Blown Apart: Crimes Against Women in Times of Conflict. London, England: Amnesty International; 2004.
17. Intitiative HH, Institute OS. Characterizing Sexual Violence in the Democratic Republic of the Congo: Profiles of Violence, Community Responses, and Implications for the Protection of Women. Cambridge, MA: Harvard University 2009.
18. Stark L, Wessells M. Sexual Violence as a Weapon of War. JAMA. 2012;308(7):677-8.

For peer review only

19. Guterres A. Conflict Related Sexual Violence: Report of the United Nations Secretary General. United Nations; 2019.
20. O'Brien M, Tolosa MX. The effect of the 2014 West Africa Ebola virus disease epidemic on multi-level violence against women. International journal of human rights in healthcare. 2016;9(3):151-60.
21. Chandan JS, Taylor J, Bradbury - Jones C, Nirantharakumar K, Kane E, Bandyopadhyay S. COVID-19: a public health approach to manage domestic violence is needed. The Lancet Public Health. 2020;5(6).
22. Mittal S, Singh T. Gender-Based Violence During COVID-19 Pandemic: A Mini-Review. Frontiers in Global Women's Health. 2020;1(4).
23. Bradbury-Jones C, Isham L. The pandemic paradox: The consequences of COVID-19 on domestic violence. J Clin Nurs. 2020;29(13-14):2047-9.
24. UNFPA. Millions more cases of violence, child marriage, female genital mutilation, unintended pregnancy expected due to the COVID-19 pandemic 2020 [Available from: https://www.unfpa.org/news/millions-more-cases-violence-child-marriage-female-genital-mutilation-unintended-pregnancies.
25. Simba H, Ngcobo S. Are Pandemics Gender Neutral? Women's Health and COVID-19. Frontiers in Global Women's Health. 2020;1(8).
26. Usher K, Bhullar N, Durkin J, Gyamfi N, Jackson D. Family violence and COVID-19: Increased vulnerability and reduced options for support
. International Journal of Mental Health Nursing. 2020;29(4).
27. Onyango M, Resnick K, Davis A, Shah R. Gender-Based Violence Among Adolescent Girls and Young Women: A Neglected Consequence of the West African Ebola Outbreak: Medical, Anthropological, and Public Health Perspectives. 2019. p. 121-32.
28. Giannini S, Albrectsen AB. COVID-19 school closures around the world will hit girls hardest UNESCO; 2020 [
29. Ethical and safety recommendations for intervention research on violence against women. Building on lessons from the WHO publication *Putting women first: ethical and safety recommendations for research on domestic violence against women*. Geneva: World Health Organization; 2016.
30. The Data Protection Act, 24 (2019).
31. McHugh ML. The chi-square test of independence. Biochem Med (Zagreb). 2013;23(2):143-9.
32. Kenya. Ministry of H, Kenya National Bureau of S, National ACC, Kenya Medical Research I, National Council for P, Development, et al. Kenya demographic and health survey, 20142015.
33. Services MoLaSPoKDoCs. Violence against Children in Kenya: Findings from a National Survey, 2019. Kenya; 2019.
34. Clayton MB, Mace B. Male Victims of Sexual Assault: Phenomenology, Psychology, Physiology. J Am Acad Psychiatry Law. 2011;39(2):197-205.
35. Hlavka HR. Speaking of Stigma and the Silence of Shame:Young Men and Sexual Victimization. Men and Masculinities. 2017;20(4):482-505.
36. Edinburgh L, Pape-Blabolil J, Harpin SB, Saewyc E. Multiple perpetrator rape among girls evaluated at a hospital-based Child Advocacy Center: Seven years of reviewed cases. Child Abuse Negl. 2014;38(9):1540-51.

For peer review only

37. Chelangat N, Kipyego E, Kimani S, Aseka L, Munyao M, Amimo S. Trapped within a lockdown: understanding sexual and gender-based violence during the Covid-19 period. . Nairobi: Professional Criminologists Association of Kenya; 2020.

For peer review only

BMJ Open

Patterns of Sexual Violence Against Adults and Children during the COVID-19 Pandemic in Kenya

Journal:	*BMJ Open*
Manuscript ID	bmjopen-2021-048636.R1
Article Type:	Original research
Date Submitted by the Author:	27-Jul-2021
Complete List of Authors:	Rockowitz, Sarah; University of Birmingham, School of Psychology<br>Stevens, Laura M.; University of Birmingham, School of Psychology<br>Rockey, James; University of Birmingham, Birmingham Business School<br>Smith, Lisa; University of Leicester, Criminology<br>Ritchie, Jessica; University of Leicester<br>Colloff, Melissa; University of Birmingham, School of Psychology<br>Kanja, Wangu; Wangu Kanja Foundation<br>Cotton, Jessica; University of Birmingham, School of Psychology<br>Njoroge, Dorothy; United States International University<br>Kamau, Catherine; Wangu Kanja Foundation<br>Flowe, Heather; University of Birmingham, School of Psychology
**Primary Subject Heading**:	Public health
Secondary Subject Heading:	Global health
Keywords:	COVID-19, PUBLIC HEALTH, Child protection < PAEDIATRICS

SCHOLARONE™
Manuscripts

I, the Submitting Author has the right to grant and does grant on behalf of all authors of the Work (as defined in the below author licence), an exclusive licence and/or a non-exclusive licence for contributions from authors who are: i) UK Crown employees; ii) where BMJ has agreed a CC-BY licence shall apply, and/or iii) in accordance with the terms applicable for US Federal Government officers or employees acting as part of their official duties; on a worldwide, perpetual, irrevocable, royalty-free basis to BMJ Publishing Group Ltd ("BMJ") its licensees and where the relevant Journal is co-owned by BMJ to the co-owners of the Journal, to publish the Work in this journal and any other BMJ products and to exploit all rights, as set out in our licence.

The Submitting Author accepts and understands that any supply made under these terms is made by BMJ to the Submitting Author unless you are acting as an employee on behalf of your employer or a postgraduate student of an affiliated institution which is paying any applicable article publishing charge ("APC") for Open Access articles. Where the Submitting Author wishes to make the Work available on an Open Access basis (and intends to pay the relevant APC), the terms of reuse of such Open Access shall be governed by a Creative Commons licence – details of these licences and which Creative Commons licence will apply to this Work are set out in our licence referred to above.

Other than as permitted in any relevant BMJ Author's Self Archiving Policies, I confirm this Work has not been accepted for publication elsewhere, is not being considered for publication elsewhere and does not duplicate material already published. I confirm all authors consent to publication of this Work and authorise the granting of this licence.

For peer review only

Patterns of Sexual Violence Against Adults and Children during the COVID-19 Pandemic in Kenya

Sarah Rockowitz[1], Laura M. Stevens[1], James C. Rockey[1], Lisa L. Smith[2], Jessica Richie[2], Melissa F. Colloff[1], Wangu Kanja[3], Jessica Cotton[1], Dorothy Njoroge[4], Catherine Kamau[3], and Heather D. Flowe[1]

University of Birmingham, School of Psychology, Birmingham, UK[1]

University of Leicester, School of Criminology, Leicester, UK[2]

The Wangu Kanja Foundation, Nairobi, Kenya[3]

United States International University, Nairobi, Kenya[4]

Keywords: Gender-based violence, sexual abuse of child, rape, Kenya, exposure to violence

Word count: 4,137

Corresponding author:
Heather D. Flowe
School of Psychology, University of Birmingham
52 Pritchatts Road, Birmingham, UK B15 2SA
h.flowe@bham.ac.uk

For peer review only

ABSTRACT

Objectives This study examined patterns of sexual violence against adults and children in Kenya during the COVID-19 pandemic to inform sexual violence prevention, protection and response efforts.

Design A prospective cross-sectional research design was used with data collected from March-August 2020.

Setting Kenya

Participants 317 adults, 224 children

Main Measures Perpetrator and survivor demographic data, characteristics of the assault.

Results Bivariate analyses found that children were more likely than adults to be attacked during the daytime (59% vs. 44%, p <.001), by a single perpetrator rather than multiple perpetrators (13% vs. 31%, p <.001), in a private as opposed to a public location (66% vs. 45%, p <.001) and by someone known to the child (76% vs. 58%, p <.001). Children were violated most often by neighbours (29%) and family members (20%), whereas adults were equally likely to be attacked by strangers (41%) and persons known to them (59%). These variables were entered as predictors into a logistic regression model that significantly predicted the age group of the survivor, $\chi^2(5, N = 541) = 53.3$, p = < .001.

Conclusions Patterns of sexual violence against adult and child survivors during the COVID-19 pandemic are different, suggesting age-related measures are needed in national emergency plans to adequately address sexual violence during the pandemic and for future humanitarian crises.

Strengths and limitations of this study

This study was conducted in partnership with frontline, Human Rights Defenders, Survivor-led organizations/Networks, Social Justice Centers using a prospective study design, which enabled the systematic and rapid study of sexual violence in Kenya during the pandemic, even though there were considerable physical distancing measures in place.

The data provide detailed information about survivors and perpetrators, including where and when incidents occurred, which enabled us to compare patterns of sexual violence in adults and children.

For peer review only

The sample was comprised of individuals who were seeking help in accessing vital services; therefore, inferences about patterns nationally in Kenya cannot be made because the data may not be representative.

Information about whether patterns of sexual violence are changing during the pandemic remains unknown because sexual violence is underreported and also more difficult to report and document during emergencies/humanitarian situations/pandemics, and there is a need for real time data collection systems that gather and analyse detailed, longitudinal information about sexual violence especially in developing countries like Kenya, where service and response infrastructure are not as robust.

For peer review only

Patterns of Sexual Violence Against Adults and Children During the COVID-19 Pandemic in Kenya

This study focuses on Kenya, a country that has a long history of SGBV, which is exacerbated during times of national crisis, such as during election periods.[1] Whilst SGBV affects women, men and children, it disrpoportionately affects women and girls, with one in three women having faced SGBV in their lifetimes worldwide.[2] Previous conflicts and disasters have led to increased gender inequality, gender-based violence, and other human rights violations, owing to disruptions in response (medical), protection, and legal services.[3] The arrival of COVID-19 in Kenya in early March 2020 marked the start of another national crisis, with more than 56,000 confirmed cases as of November 2020.[3-5] In late March, President Kenyatta issued a nation-wide curfew, with all non-essential travel banned between 7pm to 5am. Schools and non-essential businesses had to close, and travel in and out of the country was heavily restricted.[4, 5] These measures have been extended and modified multiple times, and by November 2020, the number of people who could gather in groups was still limited. Schools were also still closed, but universities opened, and air travel restrictions were lifted.[6] While the measures have undoubtedly curbed the spread of the disease, they seemed to be compromising the safety and well-being of citizens. In particular, there have been widespread reports of increases in domestic and sexual violence during the COVID-19 crisis.[5, 6]

Around the world, humanitarian crises, such as natural disasters, conflict, and disease outbreaks, are associated with changing patterns of sexual violence.[7-9] After the 2010 earthquake in Haiti, for example, the odds of an adolescent girl in Haiti being sexually abused increased by 41%.[9] Increased sexual violence occurs during conflicts, notably in Rwanda, Kosovo, and the Democratic Republic of the Congo (DRC). These crimes are especially prevalent against women and children, and attacks by multiple perpetrators are common. In the DRC, for example, nearly

For peer review only

76% of women surveyed had experiences of rape that were consistent with the attack being used as a weapon of war, and 69% of women reported experiencing gang rape, with these incidents typically being perpetuated by three perpetrators on average.[10] These findings are consistent with research conducted in the Central African Republic, Libya, and Mali, which found that multiple-perpetrator rape was commonly reported by survivors.[11]

SGBV increases during disease outbreaks, with studies reporting increases in Sierra Leone, Liberia, and Guinea during the Ebola outbreaks in West Africa in 2014-2016, and especially high increases in teenage pregnancies were reported in Sierra Leone.[12, 13] Similarly, Zika and cholera outbreaks have been linked with increased incidence of domestic violence, and reductions in funding for and access to public health services.[14] Physical distancing measures implemented during pandemics are also thought to be responsible for changing patterns and increases in violence. For instance, lockdowns and curfews mean that people must remain indoors with abusers and are unable to access outside assistance because police and vital services are unavailable, and abusers can act with impunity.[15, 16]

More research on SGBV during times of compounding crises is needed, however. SGBV is highly stigmatized, which leads to underreporting, especially in developing countries that have high levels of gender inequality. Further, it is difficult to assess whether patterns and rates of SGBV are changing during times of crisis, owing to the unavailability of nationally representative data and a lack of up-to-date and recurring data collection and a lack of data harmonization which would allow for examining SGBV trends in relation to humanitarian crises, and inform effective prevention, protection, and responses.

During the ongoing COVID-19 outbreak, several months of lockdown measures, economic challenges, health concerns, and changing global relations have increased concerns of

For peer review only

a heightened risk of SGBV. This violence during lockdown is being considered a shadow pandemic with the UN Population Fund estimating an additional 31 million cases of SGBV worldwide following 6-months isolation.[16, 17] Governments in some countries have had to create or supply alternate housing for people fleeing abusive situations, as was the case in Italy and France, with hotels being used as safe houses.[16] Social isolation policies have distinct impacts on children as well. Adolescent girls' absence from school, coupled with the lack of alternative safe spaces or shelters, has been associated with increased vulnerability to sexual violence from family members and others, including guardians, neighbours and community members.[18] As seen during the Ebola crisis, the closure of schools was associated with increased sexual violence against girls and boys, child pregnancies, and child marriage.[19]

This study prospectively investigated patterns of sexual violence perpetrated against adults and children in Kenya during the COVID-19 pandemic. We analysed data from interviews with adult survivors and the guardians of child survivors conducted by human rights defenders and members of the social justice centers during the pandemic. We focus on sexual violence because it has received less attention during the pandemic compared to physical violence. Further, research to date has not compared patterns of violence for adults and children. Doing so is critical because social isolation measures may differentially affect people in relation to age, and different measures may need to be put in place depending on the age group to prevent and respond to SGBV during COVID-19.

Based on the literature reviewed above, we predicted that there would be a greater number of women and girls violated compared to men and boys. Additionally, we anticipated there would be age-related differences in the types of locations in which sexual violence is occurring. Owing to school closures, and a lack of alternative safe spaces, we predicted that

For peer review only

children would be at a greater risk than adults during the day, and in private compared to public locations. We also compared the incidence of multiple versus single perpetrator attacks to better understand the nature of the violence occurring in relation to age. To our knowledge, no previous research has compared adults and children regarding the prevalence of violations committed by multiple perpetrators in Kenya. Hence, no age group predictions were made concerning multiple perpetrators.

METHOD

Design

A quantitative between-group prospective research design was used. The criterion variable was age group (child or adult survivor). The predictor variables included the offense characteristics displayed in Table 1, which also summarises how the variables were operationalized.

For peer review only

Table 1
Descriptions of how predictor variables were coded and operationalised

Variable	Definition
Female Survivor	Whether the survivor was female (coded as 1) as opposed to male (coded as 0)
Male Perpetrator	Whether the perpetrator was male (coded as 1) as opposed to female (coded as 0)
Daytime Attack	Whether the attack occurred in the daytime (6:00am-5:59pm; coded as 1) as opposed to at night (6:00pm-5:59am; coded as 0)
Private or Public Location	Whether the attack occurred in a private home (coded as 1) as opposed to a public location where the violation could have been witnessed or interrupted by a member of the public (coded as 0).
Private Location Type	Private locations were further subdivided into victim residence (coded as 1 for victim residence, 0 for any other location public or private); perpetrator residence (coded as 1 for perpetrator residence, 0 for any other location public or private); or other residence (coded as 1 for other residence, 0 for any other location public or private).
Multiple Perpetrator	Whether the attack was perpetrated by more than one perpetrator (coded as 1) as opposed to a singular perpetrator (coded as 0).
Known or Stranger Perpetrator	Whether the attack was perpetrated by someone known to the survivor (coded as 1) or a stranger (coded as 0)
Perpetrator Relationship Type	Perpetrator relationship type was subdivided into neighbour (coded as 1 for neighbour, 0 for any other relationship type); stranger (coded as 1 for stranger, 0 for any other relationship type); family member (coded as 1 for family member, 0 for any other relationship type); acquaintance/friend (coded as 1 for acquaintance/friend, 0 for any other relationship type); spouse/husband/boyfriend (coded as 1 for spouse/husband/boyfriend, 0 for any other relationship type); authority figure (coded as 1 for authority figure, 0 for any other relationship type); or other (coded as 1 for other, 0 for any other relationship type).

For peer review only

Participants

Participants (N=787) were survivors of sexual violence. All were residents of Kenya, living in 23 counties, and had contacted human rights defenders for assistance in obtaining vital services in the aftermath of sexual violence during the COVID-19 pandemic between March and August 2020. The human rights defenders interviewed the survivors (or their legal guardians if they were under 18) about the offense upon intake. The interview protocol was informed by World Health Organization's (WHO) ethical principles for research on SGBV and safety protocols developed by the human rights defenders for conducting their work with survivors. The survivors were aged between 7 months and 72 years (M = 21.3; SD = 9.4).

Survivors were categorized into two age groups. Following definitions provided by WHO, the child group included survivors aged 17 years and younger, whereas the adult group included survivors aged 18 years and older.[1]

After excluding cases with missing data on the predictor variables, the final sample consisted of 224 survivors in the child group and 317 in the adult group. The participants in the final sample for the child group ranged in age from 8 months to 17 years (M = 12.6, SD = 3.9), 83% were girls, and 93% were perpetrated against by men, and for the adult group, ranged in age from 18 to 72 (M = 27.1, SD = 8.1) years, 92% were women, and 96% were perpetrated against by men.

Materials

The data were obtained from records held by the human rights defenders who were assisting survivors in accessing vital services during the pandemic. They interviewed survivors about the incident and recorded information about the case on their standard intake form. They recorded the date, time, and location of the incident, and gave a free text description summarising

For peer review only

the incident. The form also had specific items to document the number of perpetrators, the relationship between the survivor and perpetrator(s), the location of the attack, and the age and gender of the survivor and perpetrator. Additionally, whilst not analysed in the current paper, any services (e.g., police, medical, safe house) the survivor had accessed were also recorded.

Procedure

Each intake form was read by two members of the research team to create the dataset. They coded the data using the criteria outlined in Table 1. If there was missing data on the form, the team read the incident summary and attempted to complete the missing information.

Ethics

The confidentiality of the data was maintained by the research team, and safety precautions were taken to minimize any risks that might cause physical harm to participants of this study. Data collection involved qualitative interviews only, and participants were offered psychological services post-interview. The Kenyan Data Protection Act (2019) was adhered to in the conduct of this research study.[20] Special attention was paid to Part IV of the Act, which notes that personal data should be processed with special attention to the privacy of the data subject, data should only be collected for specified and legitimate purposes, and that the data subject has a right to know how their data is used.[20] The data belong to the Wangu Kanja Foundation and the Sexual Violence Survivors' Network in Kenya, and permission to use the data was obtained from these organisations to conduct the analyses. The research was also approved by the Science, Technology, Engineering, and Mathematics Ethics Committee at the University of Birmingham.

Patient and Public Involvement

We relied heavily on input from civil society grassroots organisations who work on the frontlines to assist survivors in accessing vital services in the aftermath of sexual violence,

For peer review only

including the Wangu Kanja Foundation and the Sexual Violence Survivors' Network in Kenya. These organisations co-developed the research questions, the study design including the data collection instruments. They also conducted participant recruitment, data collection, and assisted with manuscript preparation. Their experience and knowledge with sexual violence in Kenya informed every aspect of the project. Their reputation within the Kenyan communities enabled survivors to disclose the incidents that occurred. The Wangu Kanja Foundation and human rights defenders would also be integral in disseminating the research findings to their networks and relevant stakeholders.

Statistical analysis

As our main analysis, we used logistic regression with age group as the dependent variable to determine which offense characteristics significantly differentiated the child and adult groups. The child age group was coded as 1 in the analysis, whereas the adult age group was coded as 0. While our data contain detailed information about each attack, we restrict our analysis to a limited number of binary variables in Table 1 as predictors. This is because there is a risk of a statistical common-support problem if we use a finer grained analysis. For example, whilst we have detailed data on where survivors were attacked, or their relationship with the perpetrator, we could not exploit this as few in the child group were attacked going to work, or by their spouse or partner. To avoid this difficulty, we used the coarser coding of relationship, known versus stranger perpetrator, and whether the attack took place in a public place or in private. This ensured that there were sufficient numbers of both children and adults in all categories. We then supplemented this analysis by tabulating the finer coding in Tables 2 and 3 .

For peer review only

Table 2
Distribution of Perpetrator Relationship to Survivor within Age Group

	Child *n*=224	Adult *n*=317
Neighbour	29%	6%
Stranger	25%	41%
Family member	20%	5%
Other	12%	16%
Acquaintance/friend	11%	12%
Spouse/husband/boyfriend	3%	15%
Authority figure	2%	6%

Table 3
Distribution of Attack Location within Age Group

	Child *n*=224	Adult *n*=317
Perpetrator's house	41%	20%
Public	28%	48%
Survivor's house	23%	23%
Other house	7%	6%
Survivor/perpetrator's house	1%	3%

We conducted preliminary analyses to identify which variables to enter into the model using Pearson's chi square tests for association. This allowed for testing whether the association between age group and each of the dichotomous variables was statistically significant. Only the variables that were significantly associated with age group were entered into the logistic regression model. To control for Type 1 errors, Bonferroni corrections were applied to the .05 alpha level (adjusted alpha = .008, with 6 variables). The strength of the relationship between the individual offense characteristics was assessed using Cramer's V, which measures the magnitude of the relationship between two categorical variables.[21] Values that fall between .41 and .60 were

For peer review only

interpreted as large, whereas values that fall between .20 and .40 were interpreted as moderate in magnitude. Values smaller than .20 were regarded as associations small in magnitude. All analyses were conducted using SPSS version 26.

Our data are freely available at: https://osf.io/b9dzp/.

RESULTS

Bivariate (chi square) analysis indicates that children compared to adults were less likely to be female and less likely to be attacked by multiple perpetrators (Table 4). Children were also more likely to be attacked in a private location, by a known perpetrator and were more likely to be attacked in the daytime. The associations between age and private location, and age and multiple perpetrators was moderately large, whereas the strength of the other associations, while statistically significant, was small in magnitude.

Table 4
Comparisons between characteristics of sexual violence against children vs. adults: bivariate analysis

Variable	Child *n*=224	Adult *n*=317	Pearson's χ^2	*p*	Cramer's V
Female victim	83%	92%	11.41	0.001	0.145
Male Perpetrator	92%	94%	1.17	0.279	0.047
Daytime Attack	59%	44%	13.18	<.001	0.156
Private versus Public Location	66%	45%	21.55	<.001	0.2
Multiple Perpetrators	13%	31%	24.2	<.001	0.212
Known versus Stranger Perpetrator	76%	58%	17.86	<.001	0.182

In the logistic regression model, the variables that were statistically significant from the bivariate analysis (chi square results) were entered as predictors (i.e., female survivor, daytime attack, private versus public location, multiple perpetrators, and known versus stranger

For peer review only

perpetrator), and the dependent variable was age group. The results are shown in Table 5. The overall model was statistically significant, $\chi^2(5, N = 541) = 53.3, p < .001$. According to Nagelkerke's R^2, 13% of the variability in age group was accounted for by the predictors in the model. Child compared to adult survivors were 1.61 times more likely to be attacked during the day, and 1.72 times more likely to be attacked in private as opposed to in public. Child compared to adult survivors were also significantly less likely (*OR*=.458) to be female, and less likely (*OR*=.528) to be attacked by multiple perpetrators.

Table 5
Outputs of logistic regression by predictor variable

	df	Estimate	SE	Wald χ^2	*p*
Female victim	1	-0.782	0.29	7.3	0.007
Daytime Attack	1	0.474	0.19	6.5	0.011
Private versus Public Location	1	0.543	0.21	6.96	0.008
Multiple Perpetrators	1	-0.638	0.27	5.77	0.016
Known versus Stranger Perpetrator	1	0.295	0.34	1.6	0.21

Tables 2 and 3 present a more detailed descriptive analysis of the child and adult cases on the relationship between the perpetrator and the victim, and the locations in which the attacks took place. As can be seen, age group was significantly associated with the relationship between the perpetrator and the survivor, $\chi^2(7, N = 541) = 107.84, p < .001$. Children were most often victimized by neighbours, followed by strangers and family members, whereas adults were most often victimized by strangers followed by other types of perpetrators (customer, community member, friend of a friend) and spouses. Age group was also significantly associated with attack location, $\chi^2 (4, N = 541) = 35.59, p < .001$. Children were most often attacked at the perpetrator's house (41% of the cases), whereas adults were most often attacked in public locations (48% of cases).

For peer review only

DISCUSSION

Summary of Key Findings

We compared patterns of sexual violence committed against adults and children in Kenya during the COVID-19 pandemic. The data arose from interviews conducted by human rights defenders with survivors and describes the experiences of 541 survivors. We found that the children in our sample were on average four years younger compared to national surveys of children in Kenya.[22, 23] Further, compared to adults, children were more likely to be attacked during the day, in private as opposed to public locations, by lone perpetrators, and by neighbours. In what follows, we discuss our findings in relation to existing research and draw implications for policy.

Comparisons to Current Literature

There were significant numbers of children in our sample, which is unsurprising, as approximately half of GBV survivors are children during humanitarian crises. However, the children in our sample were 12 years old on average, which is 4 years younger than the nationally representative samples taken pre-pandemic.[22, 23] Our sample was not nationally representative due to time and resource constraints, and it must also be noted that this violence was occurring within a particular crisis and the consequences of other crises may be different. However, it is still notable that the child survivors in our sample were younger than previous national samples have indicated. A recent study in Kenya noted that survivors who are being seen in medical settings during the pandemic appear to be younger compared to before the pandemic, speculating this is due to school closures during the pandemic.[24] Although SGBV, such as domestic violence, has been linked to cases of domestic homicide in Kenya, there were no mortalities in our study sample.[25]

For peer review only

We also found that children were 1.61 times more likely than adults to be attacked during the day. This could be attributed to the way that children and adults were spending their time during the pandemic. Because schools were closed, and there was no provision of any alternative safe spaces, children may have been often left alone or under the care or supervision of neighbours or community members, which may have made them more vulnerable to attack in some instances. Children were more likely to be attacked in private as compared to public locations. Adults in our sample were about equally likely to be attacked during the day as at night. Further, in keeping with previous research, significantly more adults were violated by multiple perpetrators in one attack compared to children.[26]

The proportion of boys in the child group was larger than the proportion of men in the adult group. This may reflect differential rates of victimization for men compared to boys, as boys are more vulnerable to assault than men due to their age. Another possible reason is that sexual violence against men compared to boys is disclosed less often. The legal definition of rape in Kenya, like many countries, requires 'vaginal penetration', which reinforces sociocultural notions that men cannot be sexually victimised.[27] Further, the tendency for people to believe that the victimisation of men is harmless, coupled with self-blame and fear on the part of victims that their community and family will react negatively towards them, discourage men from seeking help, and reporting sexual offenses to the police.[28]

The children in our sample were more likely to be violated by someone they knew than a stranger. For adults, perpetrators were most likely to be strangers, followed by neighbours and community members, and spouses. The most common perpetrators for children were neighbours, followed by strangers, and family members. Adults were violated by strangers more frequently because they were often attacked when the opportunity struck, such as while walking to or from

For peer review only

work, whereas children were violated by neighbours when they were left under their supervision due to school closures and their parents' job requirements.[29] Children were attacked by neighbours and in the perpetrators' houses at higher rates than adults. Although both groups were more likely to be violated by someone they knew as opposed to a stranger, and in both groups more than half of the perpetrators were known to the survivor, there is a high proportion of stranger compared to known assailants in both age groups. There were several instances in our data set in which neighbours invited children to use a computer or access the Internet, and then assaulted them once they were inside the neighbour's residence.

Strengths and Limitations

This research was conducted in partnership with frontline, survivor-led organisations, using a prospective study design, which are key strengths. This enabled us to study sexual violence systematically and rapidly in Kenya during the pandemic, even though there were considerable physical distancing measures in place. Further, our data are unusually rich. The data provide detailed information about survivors and perpetrators, where and when the incidents occurred, which allowed for studying patterns of sexual violence. There are also several limitations of our study to note. First, the sample was comprised of individuals who were seeking help in accessing vital services. Hence, inferences about patterns nationally in Kenya cannot be made because the data may not be representative. Further, our data do not provide information about overall sexual violence trends in our study setting. Like many countries around the world, sexual violence is underreported, and detailed, longitudinal information about sexual violence incidents are lacking. Consequently, researchers struggle to make inferences about whether patterns of violence are changing during COVID-19.[30] For example, the Demographic and Health Survey in Kenya, which is a nationally representative survey of adults conducted every

For peer review only

five years, does not gather in-depth information about violations. For example, it does not collect information about the time of day (or night) the attack occurred, if there were multiple perpetrators involved in the attack, and if the attack took place in a public or private location. Similarly, the national Violence Against Children Survey in Kenya, conducted in 2010 and 2019, does not gather in depth information. We also had to rely on second-hand accounts from guardians of sexual violence against children, owing to a lack of trained personnel and adequate resources in Kenya for interviewing children. Some children were too young to be interviewed, with the youngest victim being just seven-months old. Finally, our model better accounted for patterns of violence against adults compared to children. This is because some of the factors we analysed were more applicable to adults than children (e.g., employment, romantic relationships, being alone in public).

Recommendations and Conclusion

We urge policy makers to ensure that government COVID-19 emergency management and recovery planning adequately addresses SGBV and that minimising the risk of additional SGBV risk is integrated into national crisis policies. In particular, the results above suggest this should include the provision of adequate alternative safe spaces and shelters when schools are closed. Further, many communities have voluntarily-organised neighbourhood watch groups that are focused on security issues, and these should be explicitly expanded and supported to monitor and prevent SGBV. Community leaders have also said that there is a need for more social halls – community facilities for holding meetings, which would enable screening educational films, and other social activities. These structures can be a safe space for children and can be built using constituency development funds, which each Member of Parliament in Kenya receives to undertake projects that will address the urgent needs of their constituents.

For peer review only

Our results indicate the importance of high-quality and timely data in understanding and thus combatting SGBV. We thus recommend governments invest in real-time data collection and analysis systems to capture the evolving distribution of SGBV and to allow for the study of regional trends. Data collection would allow authorities to identify crime hot spots and violations being perpetrated by serial offenders, and to monitor the accessibility of vital services to help ensure that survivors have support. This information is crucial in designing effective interventions. For example, by knowing the location and time of attacks, responsive programmes could be put in place to engage children in other activities (e.g., drama, sports, and other educational and recreation activities) when they are not in school during the day to ensure that they are not left unattended by a responsible adult. These interventions can be low cost, with communities mobilized to create such activities with the help of university students, local NGOs, neighbourhood teachers, and religious organizations. Police patrols and community initiatives could also be planned for times at which SGBV rates peak to deter attacks and apprehend offenders. Further, the installation of street lighting might deter perpetrators from attacking women and children. Another suggestion is to establish a national sexual offender register in Kenya that would warn communities about high-risk offenders. The collection of real-time data can also inform educational programs that sensitize parents and children about community risks. These efforts must be survivor-centred, involving survivors in the implementation and evaluation of the systems.

More generally, the results in this paper highlight the latent risk of SGBV, particularly for women and girls. While its manifestation currently waxes and wanes dependent on the context, meaningful reductions in violence will require changing the narrative such that SGBV is understood to be a crime, a gross violation of human rights, and that its pre-eminent importance

For peer review only

as a determinant of physical, emotional, and mental health is reflected in national and county budgets. Funding for programming, interventions, and research should be included.

High rates of SGBV also necessitate adequate protection for the needs of survivors. To this end, the national government has approved the use of the National Government Affirmative Fund to facilitate the establishment of safe spaces/shelters in all 47 counties to ensure survivors' safety and security is safe guarded. However, advocacy is required to ensure the funds are directed appropriately.

The implementation of emergency referral pathways that enable survivors to access comprehensive care and support services should be enacted by government. Curfews and other social distancing regulations need to include SGBV response mechanisms to ensure the continued availability and accessibility of services for survivors. Further, the medico-legal response to SGBV can be strengthened by expediting restraining orders and prosecutions, and by establishing 'one-stop' centres to allow survivors to access essential services, and authorities to collect evidence, all in one location. This would also facilitate the preservation of evidence and protection of survivors to facilitate access to justice.

For peer review only

Declarations

Authors' Contributions
HF and WK conceptualized and designed the data collection methods utilized in this project. SR and LMS drafted the protocol which was then reviewed by HF, MC, JCR, and LMS. HF, SR and LMS analysed the data. HF, SR, LMS, JCR, MC, LLS, JR, WK, JC, DN, and CK contributed to and reviewed the draft version of the manuscript and approved the final version.

Non-author Contributors
The data were made possible by the efforts of the Survivors of Sexual Violence in Kenya and the Community Justice Centre who collected the information we analysed as part of their work in caring for survivors.

Competing interests
The authors declare that they have no competing interests.

Funding
This research was funded by ESRC Grant ES/T010207/1 (to H.D.F., M.F.C., J.C.R. and the Wangu Kanja Foundation) and AHRC Grant AH/T008091/1 (to H.D.F. and the Wangu Kanja Foundation), the Institute for Global Innovation, University of Birmingham (to H.D.F, and W.K.F.), and a research grant from the Ring for Peace Foundation (to L.S.).

Ethics
This research was approved by the University of Birmingham Science, Technology, Engineering, and Mathematics Ethics Committee (ERN_19-0183).

Availability of data and materials
All data generated during this study will be included in the published scoping review and will also be made available upon request.

Acknowledgements

We are grateful to the many, necessarily anonymous, human rights defenders who continue to work with survivors in difficult and dangerous conditions in Kenya.

For peer review only

References

1. Odhiambo A. “They Were Men in Uniform” Sexual Violence Against Women and Girls in Kenya’s 2017 Elections USA: Human Rights Watch 2017.
2. WHO. Violence against women who.int: World Health Organization; 2021 [
3. Health Mo. First Case of Coronavirus Disease Confirmed in Kenya health.go.ke: Ministry of Health; 2020 [
4. Ritchie H, Ortiz-Ospina E, Beltekian D, Mathieu E, Hasell J, Macdonald B, et al. Kenya: Coronavirus Pandemic Country Profile ourworldindata.org: ourworldindata.org; 2020 [
5. Flowe H, Rockowitz S, Rockey J, Kanja W. Sexual and Other Forms of Violence During the COVID-19 Pandemic Emergency in Kenya. The University of Birmingham 2020.
6. Bearak M, Ombuor R. Kenya’s coronavirus curfew begins with wave of police crackdowns. The Washington post. 2020.
7. Marsh M, Purdin S, Navani S. Addressing sexual violence in humanitarian emergencies. Glob Public Health. 2006;1(2):133-46.
8. Dara Kay C, Ragnhild N. Sexual violence in armed conflict: Introducing the SVAC dataset, 1989–2009. Journal of peace research. 2014;51(3):418-28.
9. Sloand E, Killion C, Yarandi H, Sharps P, Lewis-O'Connor A, Hassan M, et al. Experiences of violence and abuse among internally displaced adolescent girls following a natural disaster. J Adv Nurs. 2017;73(12):3200-8.
10. Intitiative HH, Institute OS. Characterizing Sexual Violence in the Democratic Republic of the Congo: Profiles of Violence, Community Responses, and Implications for the Protection of Women. Cambridge, MA: Harvard University 2009.
11. Guterres A. Conflict Related Sexual Violence: Report of the United Nations Secretary General. United Nations; 2019.
12. O’Brien M, Tolosa MX. The effect of the 2014 West Africa Ebola virus disease epidemic on multi-level violence against women. International journal of human rights in healthcare. 2016;9(3):151-60.
13. Schwartz DA, Anoko JN, Abramowitz SA. Pregnant in the Time of Ebola Women and Their Children in the 2013-2015 West African Epidemic [text]. Cham: Springer International Publishing : Imprint: Springer,; 2019.
14. Chandan JS, Taylor J, Bradbury - Jones C, Nirantharakumar K, Kane E, Bandyopadhyay S. COVID-19: a public health approach to manage domestic violence is needed. The Lancet Public Health. 2020;5(6).
15. Mittal S, Singh T. Gender-Based Violence During COVID-19 Pandemic: A Mini-Review. Frontiers in Global Women's Health. 2020;1(4).
16. Bradbury-Jones C, Isham L. The pandemic paradox: The consequences of COVID-19 on domestic violence. J Clin Nurs. 2020;29(13-14):2047-9.
17. UNFPA. Millions more cases of violence, child marriage, female genital mutilation, unintended pregnancy expected due to the COVID-19 pandemic 2020 [Available from: https://www.unfpa.org/news/millions-more-cases-violence-child-marriage-female-genital-mutilation-unintended-pregnancies.
18. Onyango M, Resnick K, Davis A, Shah R. Gender-Based Violence Among Adolescent Girls and Young Women: A Neglected Consequence of the West African Ebola Outbreak: Medical, Anthropological, and Public Health Perspectives. 2019. p. 121-32.

For peer review only

19. Giannini S, Albrectsen AB. Covid-19 school closures around the world will hit girls hardest unesco.org: UNESCO; 2020
20. The Data Protection Act, 24 (2019).
21. McHugh ML. The chi-square test of independence. Biochem Med (Zagreb). 2013;23(2):143-9.
22. Kenya. Ministry of H, Kenya National Bureau of S, National ACC, Kenya Medical Research I, National Council for P, Development, et al. Kenya demographic and health survey, 20142015.
23. Services MoLaSPoKDoCs. Violence against Children in Kenya: Findings from a National Survey, 2019. Kenya; 2019.
24. Johnson K, Green L, Volpellier M, Kidenda S, McHale T, Naimer K, Mishori R. The impact of COVID-19 on services for people affected by sexual and gender-based violence. Int J Gynaecol Obstet. 2020;150(3):285-287.
25. Mungai C. Domestic Violence in Kenya: Stop Blaming Women Cambridge, MA: Nieman Foundation; 2019.
26. Edinburgh L, Pape-Blabolil J, Harpin SB, Saewyc E. Multiple perpetrator rape among girls evaluated at a hospital-based Child Advocacy Center: Seven years of reviewed cases. Child Abuse Negl. 2014;38(9):1540-51.
27. Clayton MB, Mace B. Male Victims of Sexual Assault: Phenomenology, Psychology, Physiology. J Am Acad Psychiatry Law. 2011;39(2):197-205.
28. Hlavka HR. Speaking of Stigma and the Silence of Shame:Young Men and Sexual Victimization. Men and Masculinities. 2017;20(4):482-505.
29. Simba H, Ngcobo S. Are Pandemics Gender Neutral? Women's Health and COVID-19. Frontiers in Global Women's Health. 2020;1(8).
30. Chelangat N, Kipyego E, Kimani S, Aseka L, Munyao M, Amimo S. Trapped within a lockdown: understanding sexual and gender-based violence during the Covid-19 period. Nairobi: Professional Criminologists Association of Kenya; 2020.

For peer review only

BMJ Open

Patterns of Sexual Violence Against Adults and Children during the COVID-19 Pandemic in Kenya: A Prospective Cross-Sectional Study

Journal:	*BMJ Open*
Manuscript ID	bmjopen-2021-048636.R2
Article Type:	Original research
Date Submitted by the Author:	19-Aug-2021

Complete List of Authors:	Rockowitz, Sarah; University of Birmingham, School of Psychology<br>Stevens, Laura M.; University of Birmingham, School of Psychology<br>Rockey, James; University of Birmingham, Birmingham Business School<br>Smith, Lisa; University of Leicester, Criminology<br>Ritchie, Jessica; University of Leicester<br>Colloff, Melissa; University of Birmingham, School of Psychology<br>Kanja, Wangu; Wangu Kanja Foundation<br>Cotton, Jessica; University of Birmingham, School of Psychology<br>Njoroge, Dorothy; United States International University<br>Kamau, Catherine; Wangu Kanja Foundation<br>Flowe, Heather; University of Birmingham, School of Psychology

**Primary Subject Heading**:	Public health
Secondary Subject Heading:	Global health
Keywords:	COVID-19, PUBLIC HEALTH, Child protection < PAEDIATRICS

SCHOLARONE™ Manuscripts

BMJ

I, the Submitting Author has the right to grant and does grant on behalf of all authors of the Work (as defined in the below author licence), an exclusive licence and/or a non-exclusive licence for contributions from authors who are: i) UK Crown employees; ii) where BMJ has agreed a CC-BY licence shall apply, and/or iii) in accordance with the terms applicable for US Federal Government officers or employees acting as part of their official duties; on a worldwide, perpetual, irrevocable, royalty-free basis to BMJ Publishing Group Ltd ("BMJ") its licensees and where the relevant Journal is co-owned by BMJ to the co-owners of the Journal, to publish the Work in this journal and any other BMJ products and to exploit all rights, as set out in our licence.

The Submitting Author accepts and understands that any supply made under these terms is made by BMJ to the Submitting Author unless you are acting as an employee on behalf of your employer or a postgraduate student of an affiliated institution which is paying any applicable article publishing charge ("APC") for Open Access articles. Where the Submitting Author wishes to make the Work available on an Open Access basis (and intends to pay the relevant APC), the terms of reuse of such Open Access shall be governed by a Creative Commons licence – details of these licences and which Creative Commons licence will apply to this Work are set out in our licence referred to above.

Other than as permitted in any relevant BMJ Author's Self Archiving Policies, I confirm this Work has not been accepted for publication elsewhere, is not being considered for publication elsewhere and does not duplicate material already published. I confirm all authors consent to publication of this Work and authorise the granting of this licence.

For peer review only

Patterns of Sexual Violence Against Adults and Children during the COVID-19 Pandemic in Kenya: a prospective cross-sectional study

Sarah Rockowitz[1], Laura M. Stevens[1], James C. Rockey[1], Lisa L. Smith[2], Jessica Richie[2], Melissa F. Colloff[1], Wangu Kanja[3], Jessica Cotton[1], Dorothy Njoroge[4], Catherine Kamau[3], and Heather D. Flowe[1]

University of Birmingham, School of Psychology, Birmingham, UK[1]

University of Leicester, School of Criminology, Leicester, UK[2]

The Wangu Kanja Foundation, Nairobi, Kenya[3]

United States International University, Nairobi, Kenya[4]

Keywords: Gender-based violence, sexual abuse of child, rape, Kenya, exposure to violence

Word count: 4,796

Corresponding author:
Heather D. Flowe
School of Psychology, University of Birmingham
52 Pritchatts Road, Birmingham, UK B15 2SA
h.flowe@bham.ac.uk

For peer review only

ABSTRACT

Objectives This study examined patterns of sexual violence against adults and children in Kenya during the COVID-19 pandemic to inform sexual violence prevention, protection and response efforts.

Design A prospective cross-sectional research design was used with data collected from March-August 2020.

Setting Kenya

Participants 317 adults, 224 children

Main Measures Perpetrator and survivor demographic data, characteristics of the assault.

Results Bivariate analyses found that children were more likely than adults to be attacked during the daytime (59% vs. 44%, p <.001), by a single perpetrator rather than multiple perpetrators (31% vs. 13%, p <.001), in a private as opposed to a public location (66% vs. 45%, p <.001) and by someone known to the child (76% vs. 58%, p <.001). Children were violated most often by neighbours (29%) and family members (20%), whereas adults were equally likely to be attacked by strangers (41%) and persons known to them (59%). These variables were entered as predictors into a logistic regression model that significantly predicted the age group of the survivor, $\chi^2(5, N = 541) = 53.3, p = < .001$.

Conclusions Patterns of sexual violence against adult and child survivors during the COVID-19 pandemic are different, suggesting age-related measures are needed in national emergency plans to adequately address sexual violence during the pandemic and for future humanitarian crises.

Strengths and limitations of this study

This study was conducted in partnership with frontline, Human Rights Defenders, Survivor-led organizations/Networks, Social Justice Centers using a prospective study design, which enabled the systematic and rapid study of sexual violence in Kenya during the pandemic, even though there were considerable physical distancing measures in place.

The data provide detailed information about survivors and perpetrators, including where and when incidents occurred, which enabled us to compare patterns of sexual violence in adults and children.

For peer review only

The sample was comprised of individuals who were seeking help in accessing vital services; therefore, inferences about patterns nationally in Kenya cannot be made because the data may not be representative.

Information about whether patterns of sexual violence are changing during the pandemic remains unknown because sexual violence is underreported and also more difficult to report and document during emergencies/humanitarian situations/pandemics, and there is a need for real time data collection systems that gather and analyse detailed, longitudinal information about sexual violence especially in resource-limited countries like Kenya, where service and response infrastructure are not as robust.

For peer review only

Patterns of Sexual Violence Against Adults and Children During the COVID-19 Pandemic in Kenya

This study focuses on Kenya, a country that has a long history of SGBV, which is exacerbated during times of national crisis, such as during election periods.[1] Whilst SGBV affects women, men and children, it disproportionately affects women and girls, with one in three women having faced SGBV in their lifetimes worldwide.[2] Previous conflicts and disasters have led to increased gender inequality, gender-based violence, and other human rights violations, owing to disruptions in response (medical), protection, and legal services.[3] The arrival of COVID-19 in Kenya in early March 2020 marked the start of another national crisis, with more than 56,000 confirmed cases as of November 2020.[3-5] In late March, President Kenyatta issued a nation-wide curfew, with all non-essential travel banned between 7pm to 5am. Schools and non-essential businesses had to close, and travel in and out of the country was heavily restricted.[4, 5] These measures have been extended and modified multiple times, and by November 2020, the number of people who could gather in groups was still limited. Schools were also still closed, but universities opened, and air travel restrictions were lifted.[6] While the measures have undoubtedly curbed the spread of the disease, they seemed to be compromising the safety and well-being of citizens. In particular, there have been widespread reports of increases in domestic and sexual violence during the COVID-19 crisis.[5, 6]

Around the world, humanitarian crises, such as natural disasters, conflict, and disease outbreaks, are associated with changing patterns of sexual violence.[7-9] After the 2010 earthquake in Haiti, for example, the odds of an adolescent girl in Haiti being sexually abused increased by 41%.[9] Increased sexual violence occurs during conflicts, notably in Rwanda, Kosovo, and the Democratic Republic of the Congo (DRC). These crimes are especially prevalent against women and children, and attacks by multiple perpetrators are common. In the DRC, for example, nearly

For peer review only

76% of women surveyed had experiences of rape that were consistent with the attack being used as a weapon of war, and 69% of women reported experiencing gang rape, with these incidents typically being perpetuated by three perpetrators on average.[10] These findings are consistent with research conducted in the Central African Republic, Libya, and Mali, which found that multiple-perpetrator rape was commonly reported by survivors.[11]

SGBV increases during disease outbreaks, with studies reporting increases in Sierra Leone, Liberia, and Guinea during the Ebola outbreaks in West Africa in 2014-2016, and especially high increases in teenage pregnancies were reported in Sierra Leone.[12, 13] Similarly, Zika and cholera outbreaks have been linked with increased incidence of domestic violence, and reductions in funding for and access to public health services.[14] Physical distancing measures implemented during pandemics are also thought to be responsible for changing patterns and increases in violence. For instance, lockdowns and curfews mean that people must remain indoors with abusers and are unable to access outside assistance because police and vital services are unavailable, and abusers can act with impunity.[15, 16]

More research on SGBV during times of compounding crises is needed, however. SGBV is highly stigmatized, which leads to underreporting, especially in resource-limited countries that have high levels of gender inequality. Further, it is difficult to assess whether patterns and rates of SGBV are changing during times of crisis, owing to the unavailability of nationally representative data and a lack of up-to-date and recurring data collection and a lack of data harmonization which would allow for examining SGBV trends in relation to humanitarian crises, and inform effective prevention, protection, and responses.

During the ongoing COVID-19 outbreak, several months of lockdown measures, economic challenges, health concerns, and changing global relations have increased concerns of

For peer review only

a heightened risk of SGBV. This violence during lockdown is being considered a shadow pandemic with the UN Population Fund estimating an additional 31 million cases of SGBV worldwide following 6-months isolation.[16, 17] Governments in some countries have had to create or supply alternate housing for people fleeing abusive situations, as was the case in Italy and France, with hotels being used as safe houses.[16] Social isolation policies have distinct impacts on children as well. Adolescent girls' absence from school, coupled with the lack of alternative safe spaces or shelters, has been associated with increased vulnerability to sexual violence from family members and others, including guardians, neighbours and community members.[18] As seen during the Ebola crisis, the closure of schools was associated with increased sexual violence against girls and boys, child pregnancies, and child marriage.[19]

This study prospectively investigated patterns of sexual violence perpetrated against adults and children in Kenya during the COVID-19 pandemic. We analysed data from interviews with adult survivors and the guardians of child survivors conducted by human rights defenders and members of the social justice centers during the pandemic. We focus on sexual violence because it has received less attention during the pandemic compared to physical violence. Further, research to date has not compared patterns of violence for adults and children. Doing so is critical because social isolation measures may differentially affect people in relation to age, and different measures may need to be put in place depending on the age group to prevent and respond to SGBV during COVID-19.

Based on the literature reviewed above, we predicted that there would be a greater number of women and girls violated compared to men and boys. Additionally, we anticipated there would be age-related differences in the types of locations in which sexual violence is occurring. Owing to school closures, and a lack of alternative safe spaces, we predicted that

For peer review only

children would be at a greater risk than adults during the day, and in private compared to public locations. We also compared the incidence of multiple versus single perpetrator attacks to better understand the nature of the violence occurring in relation to age. To our knowledge, no previous research has compared adults and children regarding the prevalence of violations committed by multiple perpetrators in Kenya. Hence, no age group predictions were made concerning multiple perpetrators.

METHOD

Design

A quantitative between-group prospective research design was used. The criterion variable was age group (child or adult survivor). The predictor variables included the offense characteristics displayed in Table 1, which also summarises how the variables were operationalized.

For peer review only

Table 1
Descriptions of how predictor variables were coded and operationalised

Variable	Definition
Female Survivor	Whether the survivor was female (coded as 1) as opposed to male (coded as 0)
Male Perpetrator	Whether the perpetrator was male (coded as 1) as opposed to female (coded as 0)
Daytime Attack	Whether the attack occurred in the daytime (6:00am-5:59pm; coded as 1) as opposed to at night (6:00pm-5:59am; coded as 0)
Private or Public Location	Whether the attack occurred in a private home (coded as 1) as opposed to a public location where the violation could have been witnessed or interrupted by a member of the public (coded as 0).
Private Location Type	Private locations were further subdivided into victim residence (coded as 1 for victim residence, 0 for any other location public or private); perpetrator residence (coded as 1 for perpetrator residence, 0 for any other location public or private); or other residence (coded as 1 for other residence, 0 for any other location public or private).
Multiple Perpetrator	Whether the attack was perpetrated by more than one perpetrator (coded as 1) as opposed to a singular perpetrator (coded as 0).
Known or Stranger Perpetrator	Whether the attack was perpetrated by someone known to the survivor (coded as 1) or a stranger (coded as 0)
Perpetrator Relationship Type	Perpetrator relationship type was subdivided into neighbour (coded as 1 for neighbour, 0 for any other relationship type); stranger (coded as 1 for stranger, 0 for any other relationship type); family member (coded as 1 for family member, 0 for any other relationship type); acquaintance/friend (coded as 1 for acquaintance/friend, 0 for any other relationship type); spouse/husband/boyfriend (coded as 1 for spouse/husband/boyfriend, 0 for any other relationship type); authority figure (coded as 1 for authority figure, 0 for any other relationship type); or other (coded as 1 for other, 0 for any other relationship type).

For peer review only

Participants

Participants (N=787) were survivors of sexual violence. All were residents of Kenya, living in 23 counties, and had contacted human rights defenders for assistance in obtaining vital services in the aftermath of sexual violence during the COVID-19 pandemic between March and August 2020. The human rights defenders interviewed the survivors (or their legal guardians if they were under 18) about the offense upon intake. The interview protocol was informed by World Health Organization's (WHO) ethical principles for research on SGBV and safety protocols developed by the human rights defenders for conducting their work with survivors. The survivors were aged between 7 months and 72 years (M = 21.3; SD = 9.4).

Survivors were categorized into two age groups. Following definitions provided by WHO, the child group included survivors aged 17 years and younger, whereas the adult group included survivors aged 18 years and older.[1]

After excluding cases with missing data on the predictor variables, the final sample consisted of 224 survivors in the child group and 317 in the adult group. The participants in the final sample for the child group ranged in age from 8 months to 17 years (M = 12.6, SD = 3.9), 83% were girls, and 93% were perpetrated against by men, and for the adult group, ranged in age from 18 to 72 (M = 27.1, SD = 8.1) years, 92% were women, and 96% were perpetrated against by men.

Materials

The data were obtained from records held by the human rights defenders who were assisting survivors in accessing vital services during the pandemic. They interviewed survivors about the incident and recorded information about the case on their standard intake form (supplementary file). They recorded the date, time, and location of the incident, and gave a free

For peer review only

text description summarising the incident. The form also had specific items to document the number of perpetrators, the relationship between the survivor and perpetrator(s), the location of the attack, and the age and gender of the survivor and perpetrator. Additionally, whilst not analysed in the current paper, any services (e.g., police, medical, safe house) the survivor had accessed were also recorded.

Procedure

Each intake form was read by two members of the research team to create the dataset. They coded the data using the criteria outlined in Table 1. If there was missing data on the form, the team read the incident summary and attempted to complete the missing information.

Ethics

The confidentiality of the data was maintained by the research team, and safety precautions were taken to minimize any risks that might cause physical harm to participants of this study. Data collection involved qualitative interviews only, and participants were offered psychological services post-interview. The Kenyan Data Protection Act (2019) was adhered to in the conduct of this research study.[20] Special attention was paid to Part IV of the Act, which notes that personal data should be processed with special attention to the privacy of the data subject, data should only be collected for specified and legitimate purposes, and that the data subject has a right to know how their data is used.[20] The data belong to the Wangu Kanja Foundation and the Sexual Violence Survivors' Network in Kenya, and permission to use the data was obtained from these organisations to conduct the analyses. The research was also approved by the Science, Technology, Engineering, and Mathematics Ethics Committee at the University of Birmingham.

Patient and Public Involvement

For peer review only

We relied heavily on input from civil society grassroots organisations who work on the frontlines to assist survivors in accessing vital services in the aftermath of sexual violence, including the Wangu Kanja Foundation and the Sexual Violence Survivors' Network in Kenya. These organisations co-developed the research questions, the study design including the data collection instruments. They also conducted participant recruitment, data collection, and assisted with manuscript preparation. Their experience and knowledge with sexual violence in Kenya informed every aspect of the project. Their reputation within the Kenyan communities enabled survivors to disclose the incidents that occurred. The Wangu Kanja Foundation and human rights defenders would also be integral in disseminating the research findings to their networks and relevant stakeholders.

Statistical analysis

As our main analysis, we used logistic regression with age group as the dependent variable to determine which offense characteristics significantly differentiated the child and adult groups. The child age group was coded as 1 in the analysis, whereas the adult age group was coded as 0. While our data contain detailed information about each attack, we restrict our analysis to a limited number of binary variables in Table 1 as predictors. This is because there is a risk of a statistical common-support problem if we use a finer grained analysis. For example, whilst we have detailed data on where survivors were attacked, or their relationship with the perpetrator, we could not exploit this as few in the child group were attacked going to work, or by their spouse or partner. To avoid this difficulty, we used the coarser coding of relationship, known versus stranger perpetrator, and whether the attack took place in a public place or in private. This ensured that there were sufficient numbers of both children and adults in all categories. We then supplemented this analysis by tabulating the finer coding in Tables 2 and 3 .

For peer review only

Table 2
Distribution of Perpetrator Relationship to Survivor within Age Group

	Child<br>*n*=224	Adult<br>*n*=317
Neighbour	29%	6%
Stranger	25%	41%
Family member	20%	5%
Other	12%	16%
Acquaintance/friend	11%	12%
Spouse/husband/boyfriend	3%	15%
Authority figure	2%	6%

Table 3
Distribution of Attack Location within Age Group

	Child<br>*n*=224	Adult<br>*n*=317
Perpetrator's house	41%	20%
Public	28%	48%
Survivor's house	23%	23%
Other house	7%	6%
Survivor/perpetrator's house	1%	3%

We conducted preliminary analyses to identify which variables to enter into the model using Pearson's chi square tests for association. This allowed for testing whether the association between age group and each of the dichotomous variables was statistically significant. Only the variables that were significantly associated with age group were entered into the logistic regression model. To control for Type 1 errors, Bonferroni corrections were applied to the .05 alpha level (adjusted alpha = .008, with 6 variables). The strength of the relationship between the individual

For peer review only
For peer review only - http://bmjopen.bmj.com/site/about/guidelines.xhtml

offense characteristics was assessed using Cramer's V, which measures the magnitude of the relationship between two categorical variables.[21] Values that fall between .41 and .60 were interpreted as large, whereas values that fall between .20 and .40 were interpreted as moderate in magnitude. Values smaller than .20 were regarded as associations small in magnitude. All analyses were conducted using SPSS version 26.

Our data are freely available at: https://osf.io/b9dzp/.

RESULTS

Bivariate (chi square) analysis indicates that children compared to adults were less likely to be female and less likely to be attacked by multiple perpetrators (Table 4). Regardless, both child and adult victims were overwhelmingly female; 83% and 92%, respectively. Children were also more likely to be attacked in a private location, by a known perpetrator and were more likely to be attacked in the daytime. The associations between age and private location, and age and multiple perpetrators was moderately large, whereas the strength of the other associations, while statistically significant, was small in magnitude.

Table 4
Comparisons between characteristics of sexual violence against children vs. adults: bivariate analysis

Variable	Child *n*=224	Adult *n*=317	Pearson's χ^2	*p*	Cramer's V
Female victim	83%	92%	11.41	0.001	0.145
Male Perpetrator	92%	94%	1.17	0.279	0.047
Daytime Attack	59%	44%	13.18	<.001	0.156
Private versus Public Location	66%	45%	21.55	<.001	0.2
Multiple Perpetrators	13%	31%	24.2	<.001	0.212
Known versus Stranger Perpetrator	76%	58%	17.86	<.001	0.182

For peer review only

In the logistic regression model, the variables that were statistically significant from the bivariate analysis (chi square results) were entered as predictors (i.e., female survivor, daytime attack, private versus public location, multiple perpetrators, and known versus stranger perpetrator), and the dependent variable was age group. The results are shown in Table 5. The overall model was statistically significant, $\chi^2(5, N = 541) = 53.3$, $p < .001$. According to Nagelkerke's R^2, 13% of the variability in age group was accounted for by the predictors in the model. Child compared to adult survivors were 1.61 times more likely to be attacked during the day, and 1.72 times more likely to be attacked in private as opposed to in public. Child compared to adult survivors were also significantly less likely (*OR*=.458) to be female, and less likely (*OR*=.528) to be attacked by multiple perpetrators.

Table 5
Outputs of logistic regression by predictor variable

	df	Estimate	SE	Wald χ^2	*p*
Female victim	1	-0.782	0.29	7.3	0.007
Daytime Attack	1	0.474	0.19	6.5	0.011
Private versus Public Location	1	0.543	0.21	6.96	0.008
Multiple Perpetrators	1	-0.638	0.27	5.77	0.016
Known versus Stranger Perpetrator	1	0.295	0.34	1.6	0.21

Tables 2 and 3 present a more detailed descriptive analysis of the child and adult cases on the relationship between the perpetrator and the victim, and the locations in which the attacks took place. As can be seen, age group was significantly associated with the relationship between the perpetrator and the survivor, $\chi^2(7, N = 541) = 107.84$, $p < .001$. Children were most often victimized by neighbours, followed by strangers and family members, whereas adults were most often victimized by strangers followed by other types of perpetrators (customer, community member, friend of a friend) and spouses. Age group was also significantly associated with attack

For peer review only

location, χ^2 (4, N = 541) = 35.59, p < .001. Children were most often attacked at the perpetrator's house (41% of the cases), whereas adults were most often attacked in public locations (48% of cases).

DISCUSSION

Summary of Key Findings

We compared patterns of sexual violence committed against adults and children in Kenya during the COVID-19 pandemic. The data arose from interviews conducted by human rights defenders with survivors and describes the experiences of 541 survivors. We found that the children in our sample were on average four years younger compared to national surveys of children in Kenya.[22, 23] Further, compared to adults, children were more likely to be attacked during the day, in private as opposed to public locations, by lone perpetrators, and by neighbours. In what follows, we discuss our findings in relation to existing research and draw implications for policy.

Comparisons to Current Literature

There were significant numbers of children in our sample, which is unsurprising, as approximately half of GBV survivors are children during humanitarian crises. However, the children in our sample were 12 years old on average, which is 4 years younger than the nationally representative samples taken pre-pandemic.[22, 23] Our sample was not nationally representative due to time and resource constraints, and it must also be noted that this violence was occurring within a particular crisis and the consequences of other crises may be different. However, it is still notable that the child survivors in our sample were younger than previous national samples have indicated. A recent study in Kenya noted that survivors who are being seen in medical settings during the pandemic appear to be younger compared to before the

For peer review only

pandemic, speculating this is due to school closures during the pandemic.[24] Although SGBV, such as domestic violence, has been linked to cases of domestic homicide in Kenya, there were no mortalities captured in our study sample.[25]

We also found that children were 1.61 times more likely than adults to be attacked during the day. This could be attributed to the way that children and adults were spending their time during the pandemic. Because schools were closed, and there was no provision of any alternative safe spaces, children may have been often left alone or under the care or supervision of neighbours or community members, which may have made them more vulnerable to attack in some instances. Children were more likely to be attacked in private as compared to public locations. Adults in our sample were about equally likely to be attacked during the day as at night. Further, in keeping with previous research, significantly more adults were violated by multiple perpetrators in one attack compared to children.[26]

The proportion of boys in the child group was larger than the proportion of men in the adult group. This may reflect differential rates of victimization for men compared to boys, as boys are more vulnerable to assault than men due to their age. Another possible reason is that sexual violence against men compared to boys is disclosed less often. The legal definition of rape in Kenya, like many countries, requires 'vaginal penetration', which reinforces sociocultural notions that men cannot be sexually victimised.[27] Further, the tendency for people to believe that the victimisation of men is harmless, coupled with self-blame and fear on the part of victims that their community and family will react negatively towards them, discourage men from seeking help, and reporting sexual offenses to the police.[28]

The children in our sample were more likely to be violated by someone they knew than a stranger. For adults, perpetrators were most likely to be strangers, followed by neighbours and

For peer review only

community members, and spouses. The most common perpetrators for children were neighbours, followed by strangers, and family members. Adults were violated by strangers more frequently because they were often attacked when the opportunity struck, such as while walking to or from work, whereas children were violated by neighbours when they were left under their supervision due to school closures and their parents' job requirements.[29] Children were attacked by neighbours and in the perpetrators' houses at higher rates than adults. Although both groups were more likely to be violated by someone they knew as opposed to a stranger, and in both groups more than half of the perpetrators were known to the survivor, there is a high proportion of stranger compared to known assailants in both age groups. There were several instances in our data set in which neighbours invited children to use a computer or access the Internet, and then assaulted them once they were inside the neighbour's residence.

Strengths and Limitations

This research was conducted in partnership with frontline, survivor-led organisations, using a prospective study design, which are key strengths. This enabled us to study sexual violence systematically and rapidly in Kenya during the pandemic, even though there were considerable physical distancing measures in place. Further, our data are unusually rich. The data provide detailed information about survivors and perpetrators, where and when the incidents occurred, which allowed for studying patterns of sexual violence. There are also several limitations of our study to note. First, the sample was comprised of individuals who were seeking help in accessing vital services. Hence, inferences about patterns nationally in Kenya cannot be made because the data may not be representative. Further, our data do not provide information about overall sexual violence trends in our study setting. Like many countries around the world, sexual violence is underreported, and detailed, longitudinal information about sexual violence

For peer review only

incidents are lacking. Consequently, researchers struggle to make inferences about whether patterns of violence are changing during COVID-19.[30] For example, the Demographic and Health Survey in Kenya, which is a nationally representative survey of adults conducted every five years, does not gather in-depth information about violations. For example, it does not collect information about the time of day (or night) the attack occurred, if there were multiple perpetrators involved in the attack, and if the attack took place in a public or private location. Similarly, the national Violence Against Children Survey in Kenya, conducted in 2010 and 2019, does not gather in depth information. We also had to rely on second-hand accounts from guardians of sexual violence against children, owing to a lack of trained personnel and adequate resources in Kenya for interviewing children. Some children were too young to be interviewed, with the youngest victim being just seven-months old. Finally, our model better accounted for patterns of violence against adults compared to children. This is because some of the factors we analysed were more applicable to adults than children (e.g., employment, romantic relationships, being alone in public).

Recommendations and Conclusion

We urge policy makers to ensure that government COVID-19 emergency management and recovery planning adequately addresses SGBV and that minimising the risk of additional SGBV risk is integrated into national crisis policies. In particular, the results above suggest this should include the provision of adequate alternative safe spaces and shelters when schools are closed. Further, many communities have voluntarily-organised neighbourhood watch groups that are focused on security issues, and these should be explicitly expanded and supported to monitor and prevent SGBV. Community leaders have also said that there is a need for more social halls – community facilities for holding meetings, which would enable screening educational films,

For peer review only

and other social activities. These structures can be a safe space for children and can be built using constituency development funds, which each Member of Parliament in Kenya receives to undertake projects that will address the urgent needs of their constituents.

Our results indicate the importance of high-quality and timely data in understanding and thus combatting SGBV. We thus recommend governments invest in real-time data collection and analysis systems to capture the evolving distribution of SGBV and to allow for the study of regional trends. Data collection would allow authorities to identify crime hot spots and violations being perpetrated by serial offenders, and to monitor the accessibility of vital services to help ensure that survivors have support. This information is crucial in designing effective interventions. For example, by knowing the location and time of attacks, there can be more vigilance and awareness of SGBV against children. Additionally, this information can be used to provide further education about SGBV against children and can highlight signs to look out for of abuse. These interventions can be low cost, with communities mobilized to create such activities with the help of university students, local NGOs, neighbourhood teachers, and religious organizations. Police patrols and community initiatives could also be planned for times at which SGBV rates peak to deter attacks and apprehend offenders. Further, the installation of street lighting might deter perpetrators from attacking women and children. Another suggestion is to establish a national sexual offender register in Kenya that would warn communities about high-risk offenders. The collection of real-time data can also inform educational programs that sensitize parents and children about community risks. These efforts must be survivor-centred, involving survivors in the implementation and evaluation of the systems.

More generally, the results in this paper highlight the latent risk of SGBV, particularly for women and girls. While its manifestation currently waxes and wanes dependent on the context,

For peer review only

429 meaningful reductions in violence will require changing the narrative such that SGBV is
430 understood to be a crime, a gross violation of human rights, and that its pre-eminent importance
431 as a determinant of physical, emotional, and mental health is reflected in national and county
432 budgets. Funding for programming, interventions, and research should be included.

433 High rates of SGBV also necessitate adequate protection for the needs of survivors. To
434 this end, the national government has approved the use of the National Government Affirmative
435 Fund to facilitate the establishment of safe spaces/shelters in all 47 counties to ensure survivors'
436 safety and security is safe guarded. However, advocacy is required to ensure the funds are
437 directed appropriately.

438 The implementation of emergency referral pathways that enable survivors to access
439 comprehensive care and support services should be enacted by government. Curfews and other
440 social distancing regulations need to include SGBV response mechanisms to ensure the
441 continued availability and accessibility of services for survivors. Further, the medico-legal
442 response to SGBV can be strengthened by expediting restraining orders and prosecutions, and by
443 establishing 'one-stop' centres to allow survivors to access essential services, and authorities to
444 collect evidence, all in one location. This would also facilitate the preservation of evidence and
445 protection of survivors to facilitate access to justice.

446

447
448
449
450
451

452

For peer review only

Declarations

Authors' Contributions

HF and WK conceptualized and designed the data collection methods utilized in this project. SR and LMS drafted the protocol which was then reviewed by HF, MC, JCR, and LMS. HF, SR and LMS analysed the data. HF, SR, LMS, JCR, MC, LLS, JR, WK, JC, DN, and CK contributed to and reviewed the draft version of the manuscript and approved the final version.

Non-author Contributors

The data were made possible by the efforts of the Survivors of Sexual Violence in Kenya and the Community Justice Centre who collected the information we analysed as part of their work in caring for survivors.

Competing interests

The authors declare that they have no competing interests.

Funding

This research was funded by ESRC Grant ES/T010207/1 (to H.D.F., M.F.C., J.C.R. and the Wangu Kanja Foundation) and AHRC Grant AH/T008091/1 (to H.D.F. and the Wangu Kanja Foundation), the Institute for Global Innovation, University of Birmingham (to H.D.F, and W.K.F.), and a research grant from the Ring for Peace Foundation (to L.S.).

Ethics

This research was approved by the University of Birmingham Science, Technology, Engineering, and Mathematics Ethics Committee (ERN_19-0183).

Availability of data and materials

All data generated during this study will be included in the published scoping review and will also be made available upon request.

Acknowledgements

We are grateful to the many, necessarily anonymous, human rights defenders who continue to work with survivors in difficult and dangerous conditions in Kenya.

For peer review only

References

1. Odhiambo A. “They Were Men in Uniform” Sexual Violence Against Women and Girls in Kenya’s 2017 Elections USA: Human Rights Watch 2017.
2. World Health Organization. Violence against women who.int: World Health Organization; 2021 [Available from: https://www.who.int/news-room/fact-sheets/detail/violence-against-women.]
3. Ministry of Health. First Case of Coronavirus Disease Confirmed in Kenya: Ministry of Health; 2020 [Available from: https://www.health.go.ke/first-case-of-coronavirus-disease-confirmed-in-kenya/#:~:text=The%20Ministry%20of%20Health%20has,in%20China%20in%20December%202019.]
4. Ritchie H, Ortiz-Ospina E, Beltekian D, Mathieu E, Hasell J, Macdonald B, et al. Kenya: Coronavirus Pandemic Country Profile ourworldindata.org: ourworldindata.org; 2020 [Available from: https://ourworldindata.org/coronavirus/country/Kenya]
5. Flowe H, Rockowitz S, Rockey J, Kanja W. Sexual and Other Forms of Violence During the COVID-19 Pandemic Emergency in Kenya. The University of Birmingham 2020.
6. Bearak M, Ombuor R. Kenya’s coronavirus curfew begins with wave of police crackdowns. The Washington Post. 2020.
7. Marsh M, Purdin S, Navani S. Addressing sexual violence in humanitarian emergencies. Glob Public Health. 2006;1(2):133-46. doi: 10.1080/17441690600652787
8. Dara Kay C, Ragnhild N. Sexual violence in armed conflict: Introducing the SVAC dataset, 1989–2009. Journal of peace research. 2014;51(3):418-28. doi:10.1177/0022343314523028
9. Sloand E, Killion C, Yarandi H, Sharps P, Lewis-O'Connor A, Hassan M, et al. Experiences of violence and abuse among internally displaced adolescent girls following a natural disaster. J Adv Nurs. 2017;73(12):3200-8. doi: 10.1111/jan.13316
10. Harvard Humanitarian Initiative, Open Society Institute.. Characterizing Sexual Violence in the Democratic Republic of the Congo: Profiles of Violence, Community Responses, and Implications for the Protection of Women. Cambridge, MA: Harvard University 2009.
11. Guterres A. Conflict Related Sexual Violence: Report of the United Nations Secretary General. United Nations; 2019.
12. O’Brien M, Tolosa MX. The effect of the 2014 West Africa Ebola virus disease epidemic on multi-level violence against women. International journal of human rights in healthcare. 2016;9(3):151-60. doi: 10.1108/ijhrh-09-2015-0027
13. Schwartz DA, Anoko JN, Abramowitz SA. Pregnant in the Time of Ebola Women and Their Children in the 2013-2015 West African Epidemic [text]. Cham: Springer International Publishing : Imprint: Springer,; 2019. doi: 10.1007/978-3-319-97637-2
14. Chandan JS, Taylor J, Bradbury - Jones C, Nirantharakumar K, Kane E, Bandyopadhyay S. COVID-19: a public health approach to manage domestic violence is needed. The Lancet Public Health. 2020;5(6). doi:10.1016/S2468-2667(20)30112-2
15. Mittal S, Singh T. Gender-Based Violence During COVID-19 Pandemic: A Mini-Review. Frontiers in Global Women's Health. 2020;1(4). doi: 10.3389/fgwh.2020.00004
16. Bradbury-Jones C, Isham L. The pandemic paradox: The consequences of COVID-19 on domestic violence. J Clin Nurs. 2020;29(13-14):2047-9. doi: 10.1111/jocn.15296

For peer review only

17. UNFPA. Millions more cases of violence, child marriage, female genital mutilation, unintended pregnancy expected due to the COVID-19 pandemic 2020 [Available from: https://www.unfpa.org/news/millions-more-cases-violence-child-marriage-female-genital-mutilation-unintended-pregnancies.

18. Onyango M, Resnick K, Davis A, Shah R. Gender-Based Violence Among Adolescent Girls and Young Women: A Neglected Consequence of the West African Ebola Outbreak: Medical, Anthropological, and Public Health Perspectives. 2019. p. 121-32. doi: 10.1007/978-3-319-97637-2_8

19. Giannini S, Albrectsen AB. Covid-19 school closures around the world will hit girls hardest unesco.org: UNESCO; 2020

20. The Data Protection Act, 24 (2019). [Available from: http://kenyalaw.org/kl/fileadmin/pdfdownloads/Acts/2019/TheDataProtectionAct__No24of2019.pdf]

21. McHugh ML. The chi-square test of independence. Biochem Med (Zagreb). 2013;23(2):143-9. doi: 10.11613/BM.2013.018

22. Kenya Ministry of Health, Kenya National Bureau of Statistics, National AIDS Control Council, Kenya Medical Research Institute, National Council for Population and Developmen. Kenya demographic and health survey, 20142015.

23. Ministry of Labour and Social Protection Kenya. Violence against Children in Kenya: Findings from a National Survey, 2019. Kenya; 2019.

24. Johnson K, Green L, Volpellier M, Kidenda S, McHale T, Naimer K, Mishori R. The impact of COVID-19 on services for people affected by sexual and gender-based violence. Int J Gynaecol Obstet. 2020;150(3):285-287. doi: 10.1002/ijgo.13285

25. Mungai C. Domestic Violence in Kenya: Stop Blaming Women Cambridge, MA: Nieman Foundation; 2019.

26. Edinburgh L, Pape-Blabolil J, Harpin SB, Saewyc E. Multiple perpetrator rape among girls evaluated at a hospital-based Child Advocacy Center: Seven years of reviewed cases. Child Abuse Negl. 2014;38(9):1540-51. doi: 10.1016/j.chiabu.2014.05.008

27. Bullock CM, Mace B. Male Victims of Sexual Assault: Phenomenology, Psychology, Physiology. J Am Acad Psychiatry Law. 2011;39(2):197-205.

28. Hlavka HR. Speaking of Stigma and the Silence of Shame: Young Men and Sexual Victimization. Men and Masculinities. 2017;20(4):482-505. doi: 10.1177/1097184x16652656

29. Simba H, Ngcobo S. Are Pandemics Gender Neutral? Women's Health and COVID-19. Frontiers in Global Women's Health. 2020;1(8). doi: 10.3389/fgwh.2020.570666

30. Chelangat N, Kipyego E, Kimani S, Aseka L, Munyao M, Amimo S. Trapped within a lockdown: understanding sexual and gender-based violence during the Covid-19 period. Nairobi: Professional Criminologists Association of Kenya; 2020.

For peer review only

SURVIVORS DATA COLLECTION INTAKE SUMMARY FORM

a. Date of incident: Time of incident:
b. Age of survivor: Sex:
c. Location(s) of incident:
- Survivor's house: **Yes/No**
- Other's house: **Yes/No**
- Place of Work: **Yes/No/I don't know**
- Perpetrator's house: **Yes/No/I don't know**
- On my way to work/ On the road: **Yes/No/I don't know**
- Other location (please describe): **Yes/No/I don't know**

d. Name of the city/town/village where the incident took place:
e. Brief description of the incident:

f. Perpetrator's Information: -
- Number of perpetrator(s): - Do you know the perpetrator(s): -
- If Yes, How
- What is the gender: - Age of the perpetrator: -

g. Post Incident Information: -
- What action did the survivor take after the incident?
    a. Seek medical attention: **Yes/No**
    b. Seek counselling: **Yes/No**
    c. Report to the police: **Yes/No**
    d. Seek legal redress: **Yes/No**
    e. Protection/Safe space/Shelter: **Yes/No**

h. If the survivor sought medical attention for the incident:
    a. Did they become HIV positive due to the incident? **Yes/No/I don't know**
    b. Did you get pregnant due to the incident? **Yes/No/I don't know**
    c. Did you contract any STI's due to the incident? **Yes/No/I don't know**
    d. Were you physically injured due to the incident? **Yes/No/I don't know**

i. Was the survivor referred to the WKF? Yes/No If **YES:** Client's Ref. No:

For peer review only

STROBE Statement—Checklist of items that should be included in reports of ***cross-sectional studies***

	Item No	Recommendation
**Title and abstract**	1	(*a*) Indicate the study's design with a commonly used term in the title or the abstract 9
		(*b*) Provide in the abstract an informative and balanced summary of what was done and what was found 40-64
**Introduction**		
Background/rationale	2	Explain the scientific background and rationale for the investigation being reported 97-152
Objectives	3	State specific objectives, including any prespecified hypotheses 153-171
**Methods**		
Study design	4	Present key elements of study design early in the paper 174-177
Setting	5	Describe the setting, locations, and relevant dates, including periods of recruitment, exposure, follow-up, and data collection 190-193
Participants	6	(*a*) Give the eligibility criteria, and the sources and methods of selection of participants 190-193
Variables	7	Clearly define all outcomes, exposures, predictors, potential confounders, and effect modifiers. Give diagnostic criteria, if applicable Table 1 (line 186)
Data sources/ measurement	8*	For each variable of interest, give sources of data and details of methods of assessment (measurement). Describe comparability of assessment methods if there is more than one group 208-215, Table 1 for methods of assessment
Bias	9	Describe any efforts to address potential sources of bias NA
Study size	10	Explain how the study size was arrived at 190-206
Quantitative variables	11	Explain how quantitative variables were handled in the analyses. If applicable, describe which groupings were chosen and why 198-200, 244-255
Statistical methods	12	(*a*) Describe all statistical methods, including those used to control for confounding 244-281, 284-290
		(*b*) Describe any methods used to examine subgroups and interactions NA
		(*c*) Explain how missing data were addressed 201
		(*d*) If applicable, describe analytical methods taking account of sampling strategy 244-281
		(*e*) Describe any sensitivity analyses NA
**Results**		
Participants	13*	(a) Report numbers of individuals at each stage of study—eg numbers potentially eligible, examined for eligibility, confirmed eligible, included in the study, completing follow-up, and analysed 190-206
		(b) Give reasons for non-participation at each stage NA
		(c) Consider use of a flow diagram NA
Descriptive data	14*	(a) Give characteristics of study participants (eg demographic, clinical, social) and information on exposures and potential confounders 190-197
		(b) Indicate number of participants with missing data for each variable of interest 201
Outcome data	15*	Report numbers of outcome events or summary measures Tables 2-4
Main results	16	(*a*) Give unadjusted estimates and, if applicable, confounder-adjusted estimates and their precision (eg, 95% confidence interval). Make clear which confounders were adjusted for and why they were included NA

For peer review only

		(*b*) Report category boundaries when continuous variables were categorized 198-200
		(*c*) If relevant, consider translating estimates of relative risk into absolute risk for a meaningful time period NA
Other analyses	17	Report other analyses done—eg analyses of subgroups and interactions, and sensitivity analyses NA
**Discussion**		
Key results	18	Summarise key results with reference to study objectives 321-328
Limitations	19	Discuss limitations of the study, taking into account sources of potential bias or imprecision. Discuss both direction and magnitude of any potential bias 374-398
Interpretation	20	Give a cautious overall interpretation of results considering objectives, limitations, multiplicity of analyses, results from similar studies, and other relevant evidence 330-372
Generalisability	21	Discuss the generalisability (external validity) of the study results 400-428
**Other information**		
Funding	22	Give the source of funding and the role of the funders for the present study and, if applicable, for the original study on which the present article is based 478-481

*Give information separately for exposed and unexposed groups.

Note: An Explanation and Elaboration article discusses each checklist item and gives methodological background and published examples of transparent reporting. The STROBE checklist is best used in conjunction with this article (freely available on the Web sites of PLoS Medicine at http://www.plosmedicine.org/, Annals of Internal Medicine at http://www.annals.org/, and Epidemiology at http://www.epidem.com/). Information on the STROBE Initiative is available at www.strobe-statement.org.

For peer review only

For peer review only - http://bmjopen.bmj.com/site/about/guidelines.xhtml
